# Cross‐species transcriptomics uncovers shared responses to water deficiency in plants

**DOI:** 10.1002/tpg2.70311

**Published:** 2026-10-08

**Authors:** Francesco Punzi, Diana Lucia Zuluaga, Qiao Wen Tan, Marek Mutwil, Gabriella Sonnante, Pasquale Luca Curci

**Affiliations:** ^1^ Institute of Biosciences and Bioresources, National Research Council (CNR‐IBBR) Bari Italy; ^2^ Department of Soil, Plant and Food Sciences University of Bari Aldo Moro Bari Italy; ^3^ Bioinformatics Institute (BII), Agency for Science, Technology and Research (A*STAR) Singapore Singapore; ^4^ Department of Plant and Environmental Sciences University of Copenhagen Frederiksberg Denmark

## Abstract

Deepening our knowledge of conserved molecular mechanisms governing stress responses in plants is fundamental for identifying new core genes and processes involved in tolerance to adverse environmental conditions. For this purpose, we developed a bioinformatic pipeline to retrieve and integrate stress‐related RNA‐seq transcriptomic data from species of interest and identify core species‐specific and cross‐species conserved genes and processes involved in stress responses. We utilized this approach to investigate the molecular mechanisms underpinning drought response in *Arabidopsis thaliana*, *Triticum aestivum*, and *Solanum lycopersicum*. We found that hormone signaling, reactive oxygen species catabolism, cell wall organization, photosynthesis, and defense mechanisms were dysregulated in response to drought in at least two species. Among the identified cross‐species conserved differentially expressed genes, *PUB19*, *EDL3*, *RAB18*, and *AAP6* confirmed to play a key role in drought response. Coexpression networks identified central genes underpinning drought‐response, such as *HB‐7* in leaves and *P5CS1* in roots. Leveraging PlantConnectome, several drought‐responsive genes not classified as such in Gene Ontology were also detected, including *EULS3*, *PUB19*, *BFT*, *DAA1*, involved in carbohydrate recognition, ubiquitination, abscisic acid signaling, and membrane integrity maintenance, highlighting the need for further investigation. In conclusion, the developed approach proved effective in identifying core drought‐responsive genes and processes and has the potential to be applied to other plant species and types of stress.

## Plain Language Summary

In this study, we created a bioinformatic pipeline to retrieve and integrate transcriptomic data related to drought stress from three plant species (common wheat, tomato, and the model plant *Arabidopsis thaliana*), to identify species‐specific and conserved drought‐responsive genes. These genes were mainly involved in key biological processes, including hormone signaling, reactive oxygen species catabolism, cell wall organization, photosynthesis, and defense mechanisms. Coexpression network analysis highlighted several key genes, while additional genes were identified using the PlantConnectome database. The approach developed in our study proved effective in identifying core drought‐responsive genes and could be applied in future studies to other plant species and types of stress.

**Abbreviations**
DEGs: differentially expressed genes
DEOs: differentially expressed orthogroups
GO: Gene Ontology
GPT: generative pretrained transformer
JI: Jaccard Index
OGs: orthogroups
PC: principal component
PCA: principal component analysis
ROS: reactive oxygen species
SRA: Sequence Read Archive
TF: transcription factor
TPM: transcripts per million

## 1 INTRODUCTION

To cope with adverse environmental conditions, plants implement a wide array of physiological and molecular adjustments, some of them shared across the plant kingdom, while others are species‐specific (Pandey et al., 2015). Among abiotic stresses, drought is one of the most detrimental (Tuberosa & Salvi, 2006), unleashing a variety of responsive mechanisms including, among others, phytohormone cascades and their interactions (involving abscisic acid [ABA], salicylic acid, ethylene, jasmonic acid signal transduction cascades, and strigolactones) (Korek & Marzec, 2023; Liao et al., 2025; Sah et al., 2016), and regulation of reactive oxygen species (ROS) levels (enzymatic and nonenzymatic ROS tightly controlled scavenging activities) (Carol, 2006; Foreman et al., 2003). Moreover, drought negatively affects photosynthesis suppressing it, thereby preventing damage caused by excessive ROS production (Chaves et al., 2008; Zuluaga et al., 2023). In addition, photosynthesis level is reduced also because of the high level of stomatal closure, and indirectly through leaf shedding or growth inhibition (Chaves et al., 2008). Plants cannot indefinitely survive without synthesizing sugars. Because of this, all these mechanisms are extremely interconnected with each other, determining the extent to which plants can tolerate the adverse environmental factors. Therefore, it is essential to deepen our understanding of core mechanisms involved in the drought response, including key genes and pathways conserved across plant species.

Transcriptomics represents an invaluable source of information and, in recent years, has provided an unprecedented amount of biological knowledge, as demonstrated by the large number of RNA‐seq datasets available as specific BioProjects in the sequence read archive (SRA) generated to investigate drought stress and to characterize changes in gene expression in response to this condition. However, most of the released data were produced with a single‐species focus, thereby overlooking the additional insights that might derive from integrating biological knowledge across different species. Examples of cross‐species analyses can be found (Curci et al., 2022; Garassino et al., 2024). Bioinformatic approaches can be extremely useful for this purpose, enabling the combination of datasets from multiple independent studies. The downside of this approach is that samples from independent experiments are subjected to different technical backgrounds, including personnel, laboratory conditions, and reagents, collectively known as batch effects (Leek et al., 2010). For this reason, raw data integration is generally not recommended, as it may lead to biased results that reflect technical variability rather than true biological differences, ultimately resulting in misleading conclusions. To address this issue, statistical methods based on batch effect modeling and surrogate variable estimation, such as ComBat‐seq (Zhang et al., 2020) and SVA (Leek et al., 2012), have been developed to reduce or eliminate unwanted technical variation, while enhancing the contribution of the biological factors of interest, thereby enabling a more robust data integration.

The main goal of this study was to analyze and integrate the transcriptomic response of three different plant species to drought stress, in order to identify shared biological processes and genes dysregulated under water scarcity conditions, and as such of peculiar interest to better investigate the response to this stress. In particular, we focused on Arabidopsis (*Arabidopsis thaliana*), common wheat (*Triticum aestivum*), and tomato (*Solanum lycopersicum*), since for Arabidopsis a large number of functional gene annotations are supported by experimental evidence, while wheat and tomato represent two important crops for human nutrition, severely affected by drought stress. To accomplish our task, we developed a new pipeline aimed at curating and integrating publicly available RNA‐seq data, addressing the underlying batch effects, and performing, for each species, differential expression analyses and exploration of the most affected biological processes, either species‐specific or conserved across species. We focused on the differentially expressed genes (DEGs) conserved across the three species and functionally described them leveraging available information for *A. thaliana*, expanded with the use of new powerful GPT (generative pretrained transformer)‐assisted resources in order to enlarge the current knowledge on drought responding genes. Finally, we explored coexpression networks to identify key conserved DEGs, of relevant interest for future plant breeding applications. The pipeline we developed enables the retrieval of core genes involved in the response to drought stress, providing a framework that can be applied to any type of stress and species.

## 2 MATERIALS AND METHODS

### 2.1 Data retrieval and processing

To retrieve all drought stress transcriptomic studies and their related metadata on *A. thaliana*, *T. aestivum*, and *S. lycopersicum*, we created a pipeline, consisting of several steps. Initially, the SRA database was queried on February 2, 2025, for drought or water stress‐related transcriptomic experiments associated with *A. thaliana*, *T. aestivum*, or *S. lycopersicum*. To integrate all relevant information, the biosample_result.xml (from BioSample database), sra_result.csv, and SraRunInfo.csv (from SRA) files were retrieved and merged. A manual curation was carried out to retain (i) RNA‐seq data released by December 31, 2024, and linked to a published paper by January 31, 2025; (ii) SRA BioProjects including both control and drought stress conditions, excluding samples with spurious annotations or related to other organisms (e.g., pathogens or endophytes), mutant genotypes, or different stressors (e.g., heat stress).

The curated metadata were used to collect and reprocess the corresponding RNA‐seq BioProject samples using the LSTrAP‐Cloud pipeline (Tan et al., 2020). Briefly, RNA‐seq samples were pseudoaligned against their respective reference transcriptome (coding sequence of *A. thaliana* [TAIR10], *T. aestivum* [IWGSC], and *S. lycopersicum* [SL3.0], retrieved from Ensembl Plants database) using kallisto with default parameters, whereby reads compatible with multiple transcripts were handled through its default probabilistic abundance estimation (Bray et al., 2016). Reprocessed samples were then filtered applying default parameters (read p_pseudoaligned_threshold ≥ 40, log10(reads_pseudoaligned_threshold) ≥ 7, p_genes_mapped_threshold ≥ 40). Finally, the count and the transcript per million (TPM) expression matrices were obtained for each species. Transcript‐level abundances were then aggregated to gene level by summing the expression values of all transcript isoforms belonging to the same gene, thereby obtaining a single expression estimate per gene. The *DESeq2* (Love et al., 2014) size‐factor normalization was applied to the counts to account for differences in sequencing depth between samples.

### 2.2 Data integration and exploration

To account for the experimental variability across studies, count datasets, for each species–organ combination, were adjusted for batch effects using the ComBat_seq() function (Zhang et al., 2020), implemented in the Bioconductor package *sva* (v. 3.36.0) (Leek et al., 2012). To preserve the treatment effect when specifying the group parameter in ComBat‐seq, genes with extreme count values (>99.5th percentile for *A. thaliana* leaf samples) were filtered out prior to batch effect correction. After batch effect correction, outlier samples were excluded from downstream analyses.

Principal component analysis (PCA) was then performed, using the R package *PCAtools* (Blighe et al., 2025) to identify the factors explaining most of the variability between the samples, obtaining correlation scores between principal components (PCs) and the investigated factors (e.g., BioProject, treatment, etc.). PCA was carried out separately for each species using both ComBat‐seq adjusted and unadjusted counts, after applying the variance stabilizing transformation.

### 2.3 Differential expression and functional overrepresentation analyses

For each species and organ, DEGs were identified through the R package *DESeq2* v. 1.44.0 (Love et al., 2014), using a Benjamini–Hochberg corrected *p*‐value ≤0.05 and a |log_2_FoldChange| ≥ log_2_(1.5). Only the expressed genes were considered for this analysis, defined as those having a count value ≥10 in at least as many samples as the smallest group size. The smallest group size was defined as the minimum between the mean of control samples and the mean of stress samples per study. DEG analysis was run also on the single BioProjects. To analyze the similarity across the BioProjects, the Jaccard index (JI), defined as the ratio of the size of the intersection and the size of the union of two sets (Jaccard, 1901), was used, and the significance of the differences among groups was calculated through Shapiro–Wilk (Shapiro & Wilk, 1965) and Wilcoxon tests (Wilcoxon, 1945). To visualize the amount of overlap between the DEG sets, heatmaps were generated with the *pheatmap* package (Kolde, 2025).

Drought‐responsive biological processes in Gene Ontology (GO) were investigated, from the up‐ and downregulated genes retrieved, by means of functional enrichment analyses, via the R package *gprofiler2* (Peterson et al., 2020). To include additional genes potentially involved in drought stress, a custom gene set (“PlantConnectome drought”) was generated from PlantConnectome (S. Lim et al., 2025) using the keyword “drought” (accessed on April 28, 2025). Retrieved entries were filtered to retain only records in which either the “Resolved Source Type” or the “Resolved Target Type” was annotated as “gene identifier.” The resulting nonredundant list of Arabidopsis gene IDs was then incorporated into the GO annotation file. PlantConnectome is a GPT‐assisted webtool able to extract relationships involving genes, molecules, compartments, stresses, organs, and other plant entities, that we here used to retrieve genes in Arabidopsis associated with drought from more than 70,000 abstracts, thus allowing to recover genes from the literature that may not be classified in the GO database.

### 2.4 Orthology

To obtain orthogroups (OGs) and transfer biological information across species, OrthoFinder version 3.0 (Emms & Kelly, 2019) was used with default parameters. To identify cross‐species conserved orthologous DEGs under drought stress, the DEGs of each species were mapped to their respective OGs, and the OGs to which at least one DEG was aligned were defined as differentially expressed orthogroups (DEOs). To visualize the number of single‐species and cross‐species conserved OGs, together with the ratio of up‐ and downregulated genes, the ComplexUpset package was used (Krassowski, 2020).

### 2.5 Coexpression network analyses

Two coexpression networks were constructed in Arabidopsis for leaves and roots using *BioNERO* (Almeida‐Silva & Venancio, 2022), which implements WGCNA algorithm (Langfelder & Horvath, 2008) to perform the calculations. The TPM table was filtered to retain only the DEGs previously identified and then used as input for the network construction. Default parameters were used, that is, Spearman correlation, and genes filtered retaining only those expressed (median TPM value across samples ≥1). The lowest 𝛽 power corresponding to a scale‐free topology *R* ^2^ of at least 0.8 was chosen, that is 13 (*R* ^2 ^= 0.828) in leaves and 11 (*R* ^2 ^= 0.808) in roots (Figure S1). An enrichment analysis was then performed for each of the network modules, and those related to “response to water” were further investigated to retrieve information on the relationships between genes. Cytoscape (Shannon et al., 2003) was used to visualize the drought‐related submodules containing transcription factors (TFs), retrieved from PlantTFDB v5.0 (F. Tian et al., 2019), and their interactors. Gene names were retrieved from PLAZA 5.0 (Van Bel et al., 2022).

## 3 RESULTS

### 3.1 Retrieval and processing of drought‐stress transcriptomic data

To enable efficient transcriptomic investigation of drought stress across plant species, a novel bioinformatic pipeline to leverage publicly available RNA‐seq datasets was developed in this study (Figure 1). As a starting point, we screened the SRA database to identify all publicly available RNA‐seq datasets on three species, two dicots, including the model plant *A. thaliana* and the vegetable crop *S. lycopersicum* (tomato), and one monocot, the cereal crop *T. aestivum* (bread wheat). After applying a manual curation to discard samples with spurious annotations or not relevant to the purpose of this study (e.g., samples from a different organism or mutant line, as well as samples annotated with different stressors, even if in combination with drought), we obtained 16, 22, and 22 BioProjects, including 242, 250, and 420 samples, for Arabidopsis, wheat, and tomato, respectively. Subsequently, for each species dataset, all identified RNA‐seq samples were retrieved, and transcript abundances were quantified (see Section 2; Figure S2). The quality statistics indicated that Arabidopsis had the highest percentage (73%) of pseudoaligned reads, followed by tomato (66.2%) and wheat (63.4%). For the mean percentage of mapped genes, the same ranking was observed (Arabidopsis 66.3%, tomato 62%, wheat 57.1%). The number of studies (and related samples) that passed the quality filtering, including control and stress treatments, was 16 (201), 21 (228), and 14 (334) for Arabidopsis, wheat, and tomato, respectively (Table S1).

**FIGURE 1:**
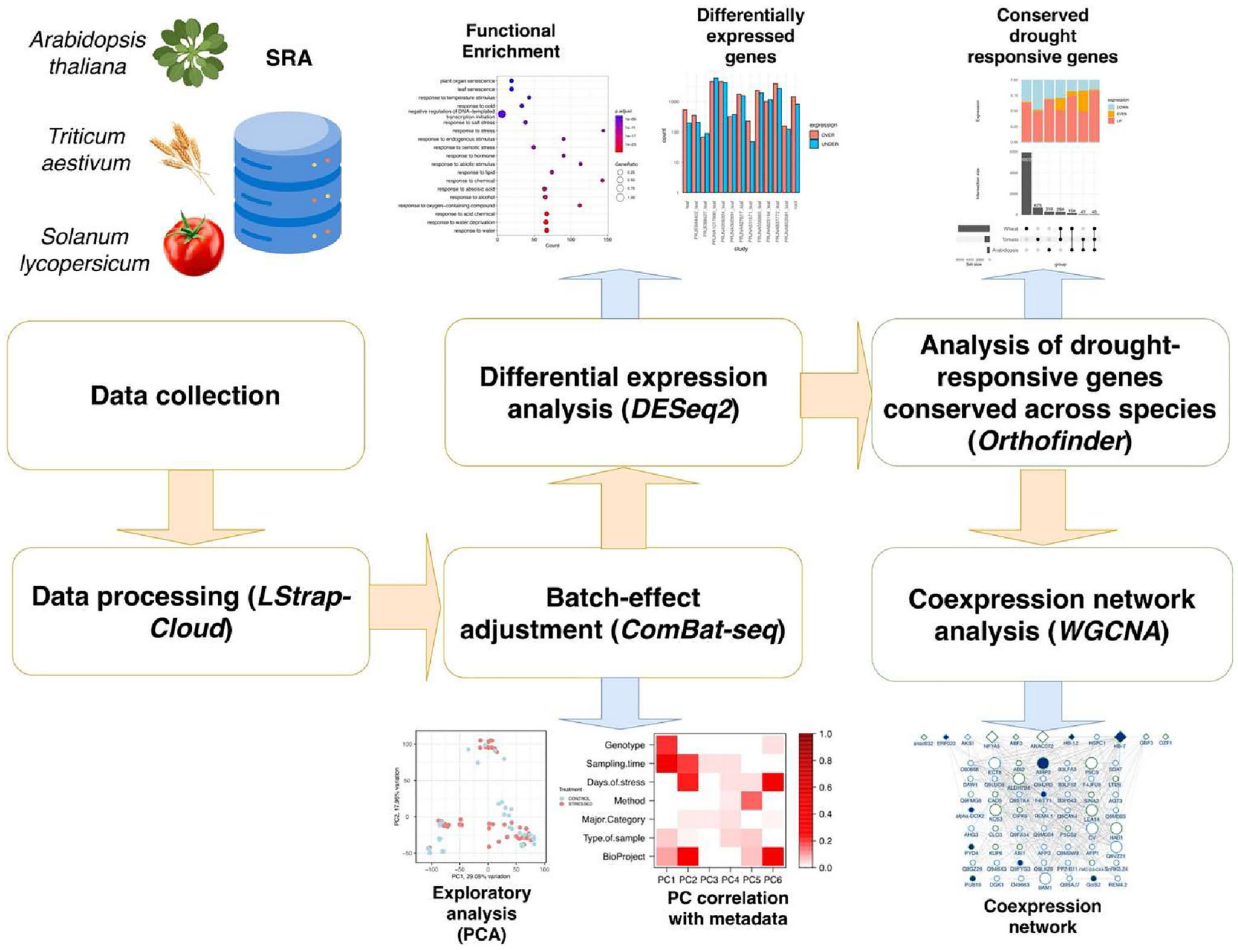
Schematic representation of the pipeline used in this study.

### 3.2 Exploratory analyses highlight high‐variability across drought‐stress studies

To explore overall data structure of the collected RNA‐seq studies, a PCA was computed for all three species under investigation (Figure S3). In all three species, transcriptomic samples did not separate based on the treatment, but mainly based on other factors, with different weights across species and PCs. For example, in Arabidopsis, PC1 variance was mostly explained by the sampling time and the days of stress, followed by the organ, while the BioProject had the strongest association on PC2. In wheat, the organ mainly drove the variance for both PC1 and PC2. In tomato, the BioProject displayed the strongest influence on PC1, while the organ was the most strongly correlated factor with PC2.

To better focus on drought stress, we applied a batch effect adjustment via *ComBat‐seq*, a well‐known tool used to mitigate the weight of experimental confounding effects and let true biological variation emerge. We focused only on leaves and roots, due to the large number of samples available. We analyzed these two organs separately, thus excluding the organ factor, a major source of variation (Figure S3). For leaves (Figure 2A–C), after the adjustment and outlier sample removal, the clustering of samples according to treatment greatly improved along PC1 in Arabidopsis and wheat, whereas in tomato, treatment significantly correlated mainly with PC2. Before the batch effect adjustment, in Arabidopsis, sampling time was the main factor explaining variance, followed by genotype and BioProject, all correlating with PC1, while PC2 was correlated with BioProject, sampling time, and days of stress. In wheat, method and sampling time had the highest correlation scores with PC1. In tomato, BioProject was the factor most strongly correlated with PC1, followed by genotype and sampling time, while PC2 had a strong correlation with sampling time and method. After the adjustment, BioProject, sampling time and genotype lost their correlation with PC1 in Arabidopsis, as did the sampling time in wheat. In tomato, the genotype, the BioProject and the sampling time completely lost their correlation with PC1 and PC2. In conclusion, after the batch effect adjustment, treatment showed an increased correlation with PC1 across all species and also with PC2 in tomato, becoming the main driver of variance.

**FIGURE 2:**
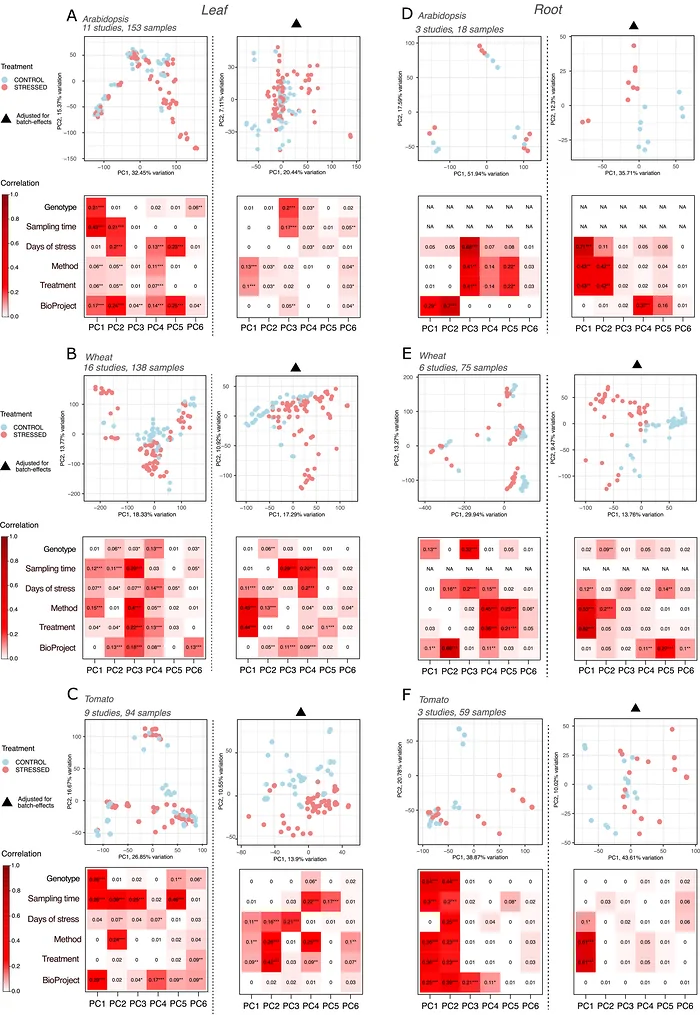
Principal component analysis (PCA) and Pearson correlation matrices between principal components (PCs) and factors of the collected and processed RNA‐seq leaf (A, B, and C) and root (D, E, and F) data colored by treatment, before (left subpanels) and after (right subpanels) batch effect adjustment for *Arabidopsis thaliana* (A and D), *Triticum aestivum* (B and E), and *Solanum lycopersicum* (C and F). Correlations are reported as *r* ^2^ values. The number of asterisks indicates the statistical significance of the correlation (*p ≤ 0.05, **p ≤ 0.01, ***p ≤ 0.001).

For roots, the batch effect adjustment resulted in similar improvements (Figure 2D–F). Before the adjustment, stressed and control samples clustered together, whereas after ComBat‐seq, a clear separation by treatment was observed. These results are confirmed by PC1 correlation scores. In Arabidopsis, the correlations of PC1 and PC2 with BioProject disappeared, whereas those with days of stress, method, and treatment increased. In wheat, treatment and method gained a very strong correlation with PC1. In tomato, before the adjustment, PC1 and PC2 were correlated with all factors, while after the adjustment, only the correlation of PC1 with treatment, method, and days of stress was maintained, with the first two being the most important factors.

### 3.3 Differential gene expression analysis

To obtain robust drought‐responsive genes in each combination of organ and species, differential expression analyses were conducted both on the sample sets obtained by the integration of all the samples from the same species and organ, adjusted for batch effect (integrated BioProject sets for each species/organ combination; Table S2), and also on the individual BioProjects (single BioProject sets for each species/organ combination). JIs were calculated to analyze the pairwise similarity among all DEG sets, separated in up‐ and downregulated, including both integrated and individual BioProject sets (Figure S4).

Considering the median number of DEGs in each combination of species, organ, and expression trend, the lowest value was observed for downregulated genes of Arabidopsis root, with 897 DEGs, while the maximum value was seen in wheat leaf upregulated genes, with 4918 genes (Figure 3A). Also, wheat was the species with the widest difference in the number of DEGs between organs, with leaves displaying 2748 more upregulated and 2471 more downregulated genes than roots. Considering the DEG numbers of the integrated sets, we obtained the lowest amount of DEGs for Arabidopsis leaf downregulated genes (199), and the highest one for wheat leaf upregulated genes (9886) (Figure 3A). A higher variability between organs was observed compared with the median DEG number obtained from individual BioProject sets, with roots having more DEGs than leaves in Arabidopsis and tomato, while wheat showed the opposite trend.

**FIGURE 3:**
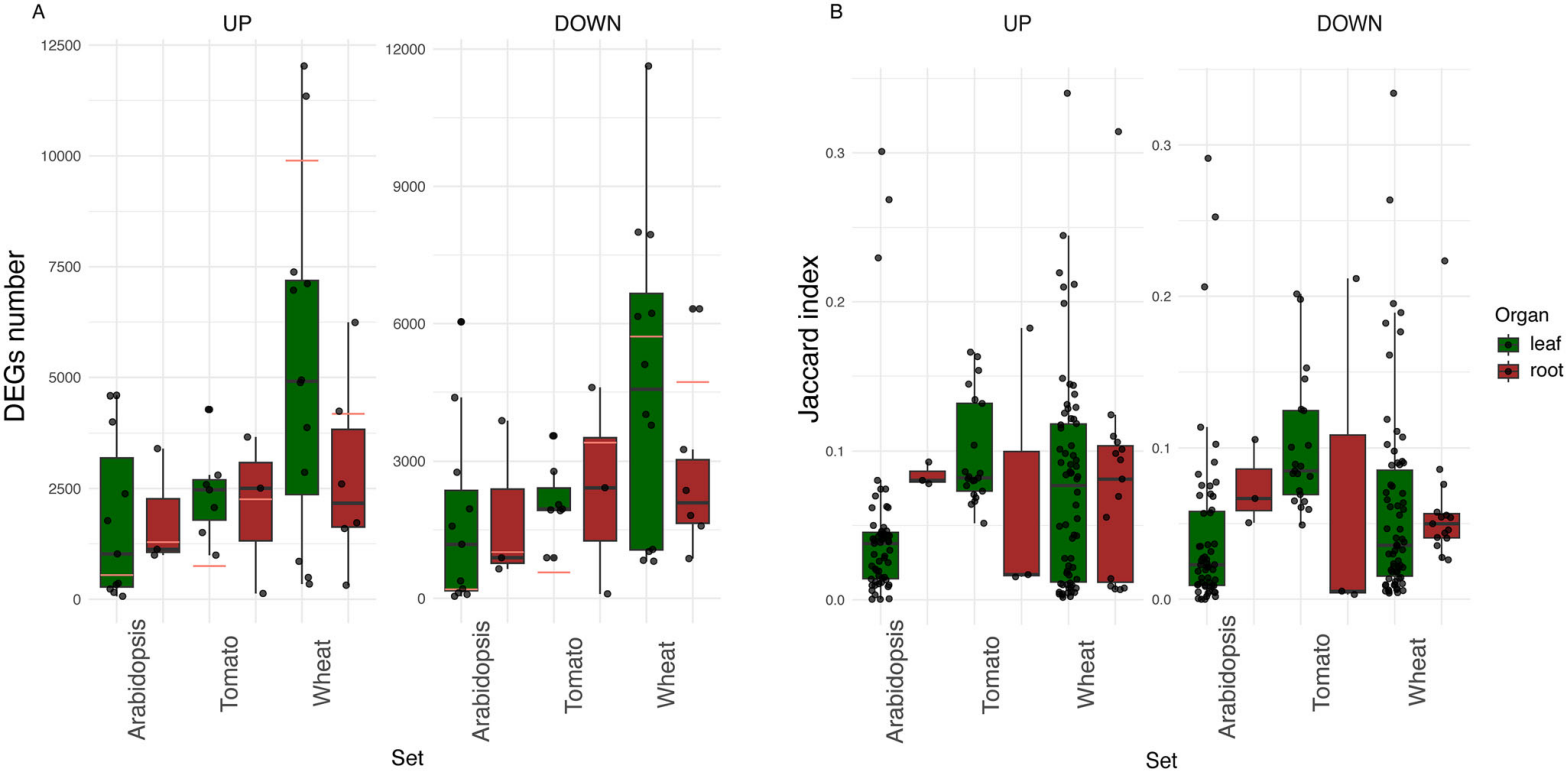
(A) Boxplots showing the distribution of differentially expressed gene (DEG) numbers identified in the individual BioProject sets for each species–organ–expression trend combination. The orange segments indicate the DEG numbers from the integrated set for the same species–organ–expression trend combination. (B) Boxplots showing the distribution of the Jaccard indexes (JIs) in all pairwise comparisons between the single BioProjects DEG sets for each species–organ–expression combination.

After calculating the JIs of the pairwise comparisons between the individual BioProject sets (Figure 3B), roots showed a higher DEG set similarity compared to leaves, both in Arabidopsis and wheat, but not in tomato. In Arabidopsis and particularly in wheat, considering the expression trend, a higher median JI was observed for upregulated DEGs compared to downregulated ones, both for leaves and roots, although not statistically significant.

Among the DEGs with the highest absolute foldchange between drought and control conditions (Table S2), we identified *DAA1*, an ABA responding AAA+‐type ATPase, *AITR1*, an ABA‐induced transcriptional repressor, *F5K20_280*, a bifunctional inhibitor/lipid‐transfer protein/seed storage 2S albumin superfamily protein, AT1G54575 (unknown function), and *LEA46*, a chaperon protein, in Arabidopsis; several ABA‐responding dehydrins and *Wcor18*, a phosphatidylethanolamine‐binding protein, in wheat; and SOLYC09G082330.2, a cupin type‐1 domain‐containing protein, *LE25*, *WAKL20*, a putative signaling receptor of extracellular matrix component, SOLYC03G116390.3, a late embryogenesis abundant protein, and SOLYC01G087180.3 (unknown function) in tomato. Several MYB TFs, mostly upregulated, were found differentially expressed in Arabidopsis, especially in roots (Table S2).

### 3.4 Enrichment and orthology analysis

To uncover the biological processes transversally involved in drought stress response across the species under investigation, up‐ and downregulated DEGs identified using the integrated BioProject sets for each combination of species and organ were used as inputs for a functional enrichment analysis (Table S3). Only processes enriched in at least two of the three species were here visualized (Figure 4A). The similarity of activated or suppressed processes across leaves and roots within species showed a higher conservation of activated processes compared to suppressed ones, with the highest overlap observed in Arabidopsis (68%) and the lowest in tomato (17%) (Table S4). At the interspecies level, pairwise comparisons also highlighted a higher conservation of activated processes compared to suppressed ones, with the only exception of the wheat‐tomato root comparison (30% for suppressed processes versus 26% for activated ones) (Table S4). Within activated processes in roots, the largest overlap was observed between Arabidopsis and wheat (52%) while the smallest between Arabidopsis and tomato (17%). In roots, all species shared 13% of the activated processes, including “response to abiotic stimulus,” “response to temperature stimulus,” and “PlantConnectome drought.” For leaves, the same ranking among the comparisons was observed with generally smaller overlap percentages (“PlantConnectome drought” the only process activated in all three species). For suppression, the major overlap was observed between wheat and tomato roots (30%), and the smallest one between Arabidopsis and tomato comparisons (4%). Only one suppressed process was shared across all three species in roots (4% of the total), that is, “response to stress.” No overlap was observed within leaves for suppressed processes in any comparison. Strikingly, “PlantConnectome drought” was found activated in all combinations of species and organ. A total of 1375, 2583, and 2246 genes were retrieved from PlantConnectome, against 424, 306, and 76 in the “response to water deprivation” Gene Ontology biological process category, and 436, 341, and 78 in the “response to water” category, in Arabidopsis, wheat, and tomato, respectively (Table S5). Among the Arabidopsis genes retrieved from PlantConnectome, 259 (18.8%) were in common with “response to water deprivation,” and 263 (19.1%) with “response to water.” Examples of DEGs associated with the enriched “PlantConnectome drought” but absent from the GO “response to water” were *EULS3*, *BFT*, *PUB19*, and *DAA1*.

**FIGURE 4:**
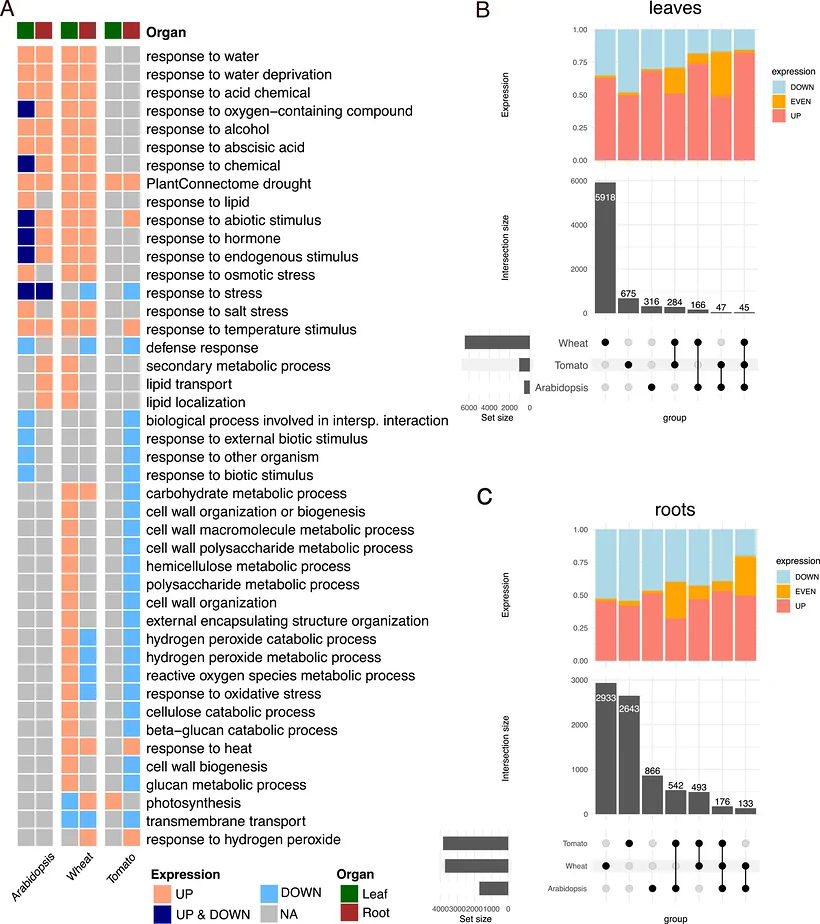
Heatmap of conserved functionally enriched Gene Ontology biological process (GO BP) terms with information about their activation and/or suppression under drought conditions in the six combinations of species and organ (processes were displayed if enriched in at least two of the three species) (A). Upset plot of differentially expressed orthogroups for each species in leaves (B) and roots (C). The stacked barplot shows the ratio of orthogroups containing a majority of upregulated genes (UP), downregulated genes (DOWN), or an even number of these (EVEN) considering the total differentially expressed genes (DEGs) of the three species.

To identify cross‐species conserved DEGs, an orthology analysis was performed (Tables S6 and S7). This analysis yielded a set of OGs, which were filtered to retain only those containing DEGs, obtaining the DEOs, separately for leaves and roots (Figure 4B,C; Table S8). Globally, 43.1% of the DEGs were mapped to species‐specific OGs, with a higher percentage in wheat (56%) compared to Arabidopsis and tomato. On the opposite, the global percentage of DEGs in shared OGs was 47.3%, with higher values in Arabidopsis and tomato (>75%) and lower in wheat (33.7%) (Table S7). The orthology results confirmed the findings from the enrichment analysis, that is, roots showed a more conserved response to drought compared to leaves across the three species (2.3% of shared OGs in roots versus 0.6% in leaves). The same trend was also observed in pairwise comparisons, where root shared DEOs accounted for 14.8% in Arabidopsis–tomato comparison, 9.7% between wheat and tomato, and 6% between Arabidopsis and wheat (Table S9). Moreover, Arabidopsis and tomato had the highest conservation of DEOs both in leaves and in roots, followed by wheat and tomato, and finally by Arabidopsis and wheat (Table S9). Looking at the expression of the genes belonging to the DEOs, upregulated genes showed a higher proportion than downregulated ones in leaves both for species‐specific DEOs (60% of upregulated OGs), and for conserved DEOs (64% of upregulated OGs), but lower in roots, with a mean of 46% in both species‐specific and conserved OGs. Among the cross‐species conserved DEGs in leaves with the highest mean foldchange across the three species, we identified *PUB19*, a U‐box E3 ligase; *EDL3*, an EID1‐like F‐box protein involved in ABA signaling; and AT4G36700 (still uncharacterized), upregulated, and *COR413‐PM4*, modulated by sugars, and *AAP6*, an amino acid transporter, downregulated. In roots, we identified *RAB18*, a dehydrin, *CBSX4*, a cystathionine β‐synthase (CBS) domain containing protein involved in the activation of thioredoxins, *T1G11.19*, part of the AWPM‐19 family, upregulated, *GDPD2*, involved in root architecture, and *FLOT1*, a membrane microdomain‐associated protein, downregulated.

### 3.5 Coexpression network analysis reveals shared drought‐responsive modules

To further investigate cross‐species conserved patterns among the DEGs identified, a coexpression network was constructed using the best annotated model species Arabidopsis, focusing on leaves and roots, separately (Figure S5A). For leaves, a total of six modules were identified, including from 34 to 134 genes, while in roots 25 modules were found, spanning from 31 to 385 genes. An enrichment analysis on each network module was carried out to gain insights into the underlying biological meaning. Among the identified modules, the black and green modules in leaves and the darkorange module in roots were the ones enriched in processes related to water stress response, such as “response to water,” “response to water deprivation,” and “PlantConnectome drought,” along with other processes such as “response to stress” and “response to abiotic stimulus” (Figure S5B).

Considering the other modules identified in leaves and their most related biological processes, the gray module was highly enriched for interaction with other organisms; the red one for biosynthesis of glycosyl compounds; and the yellow one for a variety of processes including response to chemicals, endogenous stimulus, oxygen containing compounds, hormones, and stresses, such as hypoxia. For roots, the black module was enriched for hydrogen peroxide–mediated signaling pathway; the blue one for oxygen‐related processes; the brown one for response to acid chemical; the cyan one for vitamin B6 metabolism; the dark gray one for responses to hormones, stresses and detoxification; the darkolivegreen one for amino acid metabolism; the dark turquoise one for glycosyl compounds metabolism; the gray one for responses to cold, heat, and hydrogen peroxide; the light cyan one for glucosinolate transport; the orange one for secondary metabolite metabolism; the saddlebrown for response to hypoxia; the salmon one for response to toxic substance and secondary metabolite metabolism; the tan one for phloem and xylem histogenesis; the violet one for zinc transmembrane transport; and the yellow one for cell wall organization and biogenesis (Figure S5B).

The leaf black and the root darkorange modules showed the most significant enrichment for water‐related processes and therefore we further investigated these modules (Figure 5; Table S10). Because of its large size, the black module was filtered to retain only hub genes, TFs, and their immediate neighbors to examine which of these genes were conserved DEGs across the three species (Figure 5A). In leaves, among the 11 TFs identified, *ERF023*, *HB‐7*, and *HB‐12* were differentially expressed in all three species (Figure 5A; Table S8), *ANAC072*, *NFYA5*, and *HB‐7* were hub genes in the module (Table S10), and eight were present in the PlantConnectome drought set (Figure 5A). Other noteworthy genes were *ABF3*, AT2G38820 (*DUF506*), and AT1G67300, a putative carbohydrate transporter membrane protein. We found *ERF023* upregulated in Arabidopsis and coexpressed with *GOLS2*, *MTP9*, *REM4.2*, *PYD4*, *REM4.1*, *NIPA2*, and *MMG4.18*. The TFs *HB‐7* and *HB‐12* were coexpressed with several genes, such as *LEA14*, *ALDH7B4*, *AFP1*, *HAB1*, and *AIRP2*, suggesting potential regulatory relationships with these genes. In roots, among the two TFs identified, one was downregulated and conserved across species (*FBH4*, a BHLH TF) (Figure 5B). Other important genes were the hub genes *RAB18* and *DAA1*, conserved across species, and the hub gene *P5CS1*, present in the PlantConnectome set, beyond other several conserved DEGs and hub genes, some of which have not yet been characterized.

**FIGURE 5:**
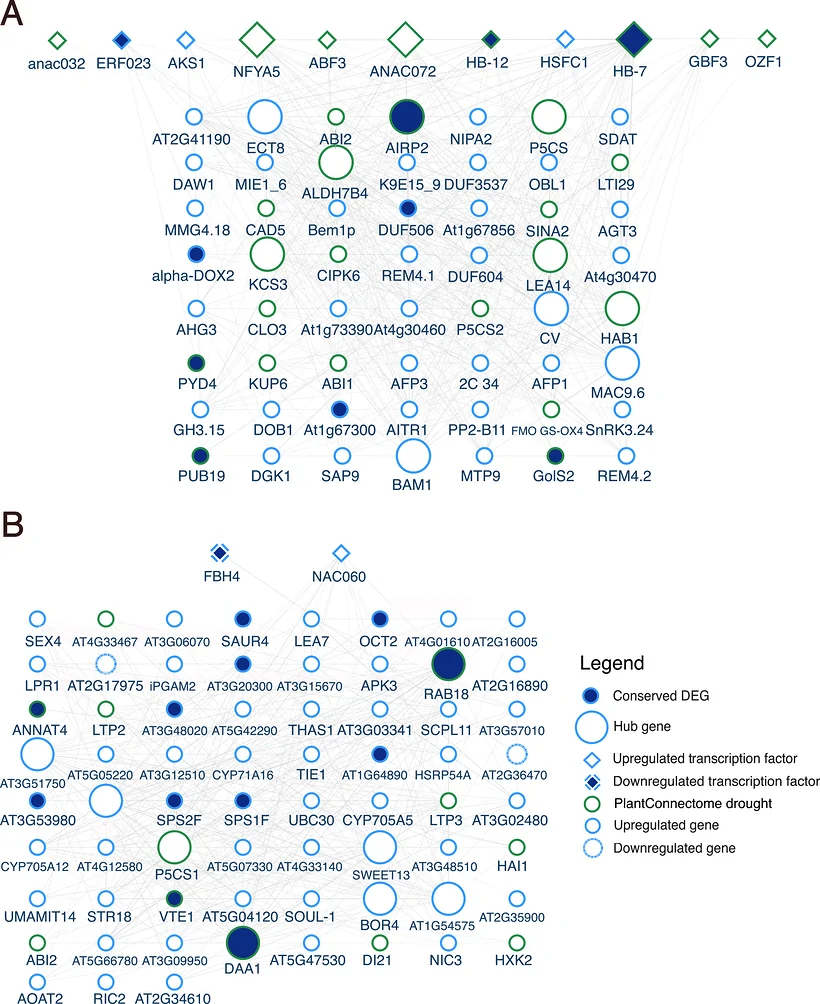
Coexpression network modules involved in “response to water.” Black module from leaves (A) and darkorange module from roots (B). A big node size encodes hub genes in the module, the night blue filling indicates differentially expressed gene (DEG) conservation across species, the green border marks genes present in the PlantConnectome drought set. Continuous node borders indicate upregulated genes, while dashed borders downregulated ones. Diamond shapes encode transcription factors. Line width represents the strength of the coexpression between two genes.

## 4 DISCUSSION

Abiotic stresses, including drought, heat, and salinity, represent major constraints to crop production by severely impairing plant growth, development, and productivity. Therefore, elucidating the physiological and molecular mechanisms underlying plant adaptation and tolerance to these adverse environmental conditions is essential for improving crop resilience and ensuring sustainable agricultural production. The aim of this study was to identify drought‐responsive biological processes and conserved genes across species through an integrative transcriptomic analysis. Uncovering the molecular mechanisms shared by different plant species in response to drought may provide valuable insights into the core components of stress tolerance and facilitate the development of strategies to enhance crop resilience (Chaddad et al., 2023; Murtaza et al., 2025). To accomplish this task, we integrated publicly available RNA‐seq data from drought‐stress studies in three plant species: the dicots Arabidopsis and tomato, and the monocot bread wheat. Arabidopsis is an important model organism, for which a vast amount of biological information is available, while tomato and wheat are two crops of remarkable importance from an agronomic and commercial standpoint.

In recent years, a growing number of RNA‐seq‐based transcriptomic studies have been conducted, leading to an unparalleled amount of biological data, freely accessible on public repositories. However, this wealth of biological information comes often with poor sample annotation (i.e., mis‐specified organ, species) requiring, as in the present study, further manual curation by the users. In parallel, new strategies have been developed to efficiently integrate sequencing data, with the aim of extracting more robust and meaningful conclusions (Chaddad et al., 2023; Ding et al., 2025; Sreedasyam et al., 2023). One of the difficulties is often represented by the significant amount of variability across studies, due for example to the biological material analyzed or to the adopted experimental conditions. Batch effects, that is, technical differences across the experiments due to factors like laboratory operators, laboratory conditions, and the instruments used, can negatively affect data integration, leading to biologically inconsistent conclusions (Zhang et al., 2020). Moreover, factors such as the genotype, developmental stage, stress conditions, and other experimental variables can introduce considerable inter‐sample variability, further complicating data integration (Ginzburg et al., 2022; Großkinsky et al., 2015).

In our study, PCA highlighted the complexity of integrating different RNA‐seq studies. Before batch‐effect correction, drought treatment was largely masked by other experimental factors, as previously mentioned (i.e., BioProject, genotype, organ, sampling time, method, and days of stress), leading to an unexpected sample clustering. However, the batch effect adjustment efficiently mitigated the artifact differences across studies, eventually unhiding the treatment factor, thus proving effective in recovering and prioritizing the authentic biological effects of drought treatment (Azad et al., 2024). It has to be noted that, although ComBat‐seq substantially reduced study‐related technical variation, only the treatment factor was explicitly preserved in the adjustment model. Other biological factors, including genotype and developmental stage, were not explicitly modeled and therefore their associated variation was not specifically protected during adjustment. Consequently, biologically meaningful variation associated with factors not explicitly modeled may have been partially attenuated during batch correction. However, the limited overlap among the DEG sets identified in the individual BioProjects suggests that substantial residual biological variability across studies remained. The corrected data should therefore be interpreted primarily as highlighting drought‐responsive transcriptional patterns shared across independent studies.

From the differential expression analysis, we identified several ABA‐responding genes involved in drought response, such as *DAA1* and *AITR1* (Shahsavari et al., 2026; H. Tian et al., 2017), both here upregulated in leaves and roots in Arabidopsis; *RAB18* (Johnson et al., 2017), an upregulated dehydrin in leaves and roots of wheat; and *LE25*, upregulated in tomato roots, and previously observed to be ABA‐induced under drought conditions in tomato (Kahn et al., 1993). Other important DEGs found are *DREB2A*, a TF binding to C‐repeat/DRE elements and involved in heat and drought stress tolerance (Sakuma et al., 2006); *LEA46*, a chaperone upregulated in Arabidopsis leaves and activated by its N‐terminal adopting an α‐helix folding (Cuevas‐Velazquez et al., 2016; Olvera‐Carrillo et al., 2010); several MYB genes, a gene family known to be involved in the response to drought stress (Blanco et al., 2022; X. Wang, Wei, et al., 2025); NAC genes, functioning as a promoter of drought tolerance at the seedling stage by regulating the expression of LEA protein gene *LEA1‐2B* (Chen et al., 2025); and *WAKL20*, upregulated in tomato roots, belonging to cell wall‐associated kinase family (WAK) known to link the extracellular matrix and the cytoplasm (Verica & He, 2002).

Several abiotic stress‐response processes were enriched. Indeed, plants rely on similar biological pathways for coping with different environmental conditions (Demmig‐Adams et al., 2022). We observed a shared activation of “response to hormone” and “response to abscisic acid” in Arabidopsis and wheat leaves and roots. This activation is also a key factor for modulating metabolism under drought conditions, as already observed in previous studies, highlighting how the activation of the ABA cascade promotes tolerance to water deficit (C. Lim et al., 2022). ABA is referred to as the stress hormone, having a central role in the regulation of metabolism under several stress conditions (Sah et al., 2016). During drought, ABA lowers the transpiration rate and promotes the expression of other drought‐responsive genes and the synthesis of secondary metabolites, such as phenylpropanoids (Y. Wang, Tong, et al., 2025). Moreover, ABA and strigolactone biosynthesis are interconnected through the carotenoid pathway, with *ERF61* suggested as a potential regulator of the cross‐talk between the two hormones through the regulation of genes involved in the carotenoid biosynthesis (Korek et al., 2025). Their interaction may enhance drought tolerance through ABA‐mediated stomatal closure (Korek & Marzec, 2023). *ERF61* may also provide a link between these hormonal pathways and ethylene signaling. Ethylene is also known to promote drought tolerance by inducing antioxidant enzyme transcription and proline synthesis, showing that hormone signaling and ROS metabolism are closely interconnected (Du et al., 2023; Shrestha et al., 2023; Yang et al., 2021). Accordingly, we found the ROS metabolism enriched in tomato and wheat, strengthening the relationship with hormone signaling. On the other hand, “defense response” was found enriched among downregulated genes in the three species, suggesting the suppression of defense pathways. For example, *WAKL20*, a negative regulator of plant immunity that inhibits resistosome assembly (Zhong et al., 2025), was here upregulated in tomato. Another conserved enriched process obtained was “photosynthesis”, which is known to be inhibited under drought stress conditions to avoid oxidative damage to the photosynthetic machinery (Chaves et al., 2008). However, we found it enriched among downregulated genes in wheat leaves, and among upregulated genes in wheat roots and tomato leaves. This may be due to differences in photosynthesis regulation under drought. In energy‐limited ecosystems, plants can also increase their photosynthetic rate during spring droughts (Miller et al., 2023). Processes related to cell wall organization and the biosynthesis of components, such as hemicellulose, were also found enriched in tomato and wheat, in accordance with previous observations of cell wall thickening in maize roots to promote drought tolerance (Han et al., 2024).

When considering the enriched “PlantConnectome drought” category, the number of associated genes was substantially higher than those identified for the GO term “response to water” across all species and organs, with the most pronounced difference observed in tomato. As such, the low number of drought‐responding genes of “response to water” and “response to water deprivation” categories in tomato suggests the importance and usefulness of the “PlantConnectome drought” set for functional studies in this species. PlantConnectome, an artificial intelligence–driven tool that retrieves functional relationships between biological entities, successfully extracted additional drought‐responsive genes that might be considered within the standard GO categories to expand them and allow discovering new biological insights. Among the genes retrieved with PlantConnectome but absent in the drought‐related GO categories of Arabidopsis, we identified *EULS3*, a lectin involved in the response to abiotic stresses (Van Hove et al., 2014); *BFT* (*BROTHER OF FT AND TFL1*), a *TFL1*‐like gene involved in flowering repression, belonging to the FT/TFL1 family that regulates the transition between vegetative and reproductive development (Moraes et al., 2019); *PUB19*, that negatively regulates drought stress (Liu et al., 2011); and *DAA1*, responding to ABA and drought‐responsive ATPase potentially involved in protein remodeling (Shahsavari et al., 2026).

From the orthology analysis, we were able to associate DEGs to DEOs. The highest DEO overlap was found between Arabidopsis and tomato, as expected, both being dicotyledons, as opposed to process conservation, where the overlap between these two species ranked last. This might be due to a lower level of completeness of tomato GO annotation (as hypothesized previously for the functional enrichment analyses) compared to the other two species here under investigation. Wheat was the species with the highest number of DEGs and species‐specific DEOs, likely due to both its hexaploid genome and the widest genetic distance present between wheat and the dicotyledons compared to the genetic distance between the two dicotyledons (Paterson et al., 2004). The conserved genes with the highest absolute value of foldchange were found in roots. Numerous ABA‐responding proteins were obtained, such as *PUB19*, a U‐box E3 ligase found upregulated in leaves modulating drought stress response (Liu et al., 2011; Seo et al., 2016); *EDL3* (EID1‐like F‐box protein 3), an F‐box protein, upregulated in leaves, part of a ubiquitin ligase involved in ABA regulation, controlling the response to drought and other abiotic stresses (Koops et al., 2011); and *RAB18* (Johnson et al., 2017), an ABA‐responsive dehydrin upregulated in roots. We also identified several membrane proteins, such as *AAP6* (amino acid permease 6), an amino acid‐proton symporter downregulated in leaves (Rentsch et al., 1996); *T1G11.19*, an AWPM‐19‐like family protein, upregulated in roots, whose rice homolog *OsPM1* functions as a plasma membrane ABA influx carrier, promoting tolerance to drought and other stresses (Koike et al., 1997; Yao et al., 2018); and *FLOT1*, downregulated in roots and associated with membrane microdomains, important for signal transduction and defense against pathogens (M. Yu et al., 2017), supporting the suppression of defense processes previously reported in all three species. Another interesting gene is *GDPD2*, downregulated in roots, regulating auxin and gibberellin signaling. Under phosphorus deficiency in soybean, it is upregulated, promoting root cell division and thus tolerance to phosphorus deficiency (Hu et al., 2024). Therefore, we suggest that *GDPD2* may be regulated in the opposite way under water deficiency.

The coexpression network allowed to uncover TF‐gene coexpression relationships, highlighting cross‐species conserved drought‐responsive genes, within the Arabidopsis system. Although WGCNA is commonly applied to genome‐wide expression datasets, the present analysis was intentionally restricted to DEGs to identify drought‐responsive coexpression modules. Consequently, the inferred network represents only the drought‐responsive transcriptome and may exclude regulators whose expression is not significantly altered, as well as those those specific to particular genotypes or experimental conditions. Therefore, the identified hub genes and TFs should be considered candidate regulators rather than a comprehensive representation of the global drought regulatory network. Among the most interesting TFs identified, *ERF023*, an ethylene‐responding TF, was previously found participating in the regulation of carotenoid metabolism in grapefruit by modulating the expression of several carotenogenic genes, including *GGPPS2*, *PDS*, *Z‐ISO*, *ZDS2/7*, *CRTISO3*, *CYP97A*, *CHYB*, *ZEP2*, and *CCD1‐2*. *ERF023* is also known to activate *PpNCED2/3* transcription in peach, promoting ABA biosynthesis (Huang et al., 2022; X. Wang et al., 2019). Among the genes coexpressed with *ERF023*, *GOLS2* was also found conserved across the studied species. It encodes galactinol synthase 2, which promotes tolerance to drought, salinity and other environmental conditions by increasing the level of osmoprotectants, such as raffinose and galactinol (Nishizawa et al., 2008; Taji et al., 2002). Other relevant genes in the leaf network were *HB‐7* and *HB‐12*, two homeobox‐leucine zipper proteins. They are TFs known to be induced under drought conditions in Arabidopsis, in particular through the ABA signaling pathway (Lee et al., 2001; Olsson et al., 2004; Söderman et al., 1996), analogously to one of their predicted interactors, *AIRP2* (Cho et al., 2011). Another *HB‐7* and *HB‐12* interactor, *LEA14*, is expressed under different abiotic stressors, including drought, and belongs to the LEA protein family, whose members have been proposed to protect cellular components and maintain membrane stability and integrity under dehydration stress (Singh et al., 2005). In roots, *FBH4* (*bHLH130*), a bHLH TF, showed conserved downregulation, and in other species its suppression promoted tanshinone biosynthesis through the gibberellin signaling, and also the chalcone synthase biosynthesis, increasing flavonoid levels and thus improving nitrogen uptake (X. Wang et al., 2023; H. Yu et al., 2026); *RAB18* and *DAA1*, two conserved DEGs previously described, emerged as hub genes in the network, further supporting their involvement in drought tolerance; *P5CS1*, a hub gene, has a key role in proline synthesis, thus being extremely important under osmotic stress (Savouré et al., 1995).

In conclusion, we have designed a new pipeline for the integrative analysis of transcriptomic data starting from a large number of collected RNA‐seq datasets, to leverage multiple studies and identify core processes and genes involved in specific stresses in any plant species. After applying this methodology to drought stress, we have gained insights into several core processes and genes involved in the response to this condition, both species‐specific and conserved across Arabidopsis, wheat and tomato. Moreover, PlantConnectome emerged as a powerful tool for expanding the GO functional enrichment, allowing to enlarge gene functional information and transfer new biological knowledge across species. We identified numerous genes that have not been experimentally characterized yet and inferred novel relationships among drought‐responsive genes, highlighting the potential to advance current knowledge on plant genes involved in drought tolerance at both the species‐specific and cross‐species levels.

## AUTHOR CONTRIBUTIONS

**Francesco Punzi**: Data curation; formal analysis; investigation; software; validation; visualization; writing—original draft. **Diana Lucia Zuluaga**: Data curation; investigation. **Qiao Wen Tan**: Resources; writing—review and editing. **Marek Mutwil**: Resources; writing—review and editing. **Gabriella Sonnante**: Funding acquisition; project administration; supervision; writing—review and editing. **Pasquale Luca Curci**: Conceptualization; funding acquisition; investigation; methodology; project administration; resources; supervision; validation; writing—original draft; writing—review and editing.

## CONFLICT OF INTEREST STATEMENT

The authors declare no conflicts of interest.

## Supporting information




**Supplemental Figure S1**. Plots showing the 𝛽 power on the x‐axis and the scale free topology R^2^ (**A**) and k mean connectivity (**B**) on the y‐axis, for leaves and roots.
**Supplemental Figure S2**. Alignment statistics for *A. thaliana* (**A‐B**), *T. aestivum* (**C‐D**) and *S. lycopersicum* (**E‐F**). The red dotted lines indicate the utilised thresholds, i.e. read p_pseudoaligned_threshold ≥ 40, log10(reads_pseudoaligned_threshold) ≥ 7, p_genes_mapped_threshold ≥ 40. The top‐right area contains the samples that passed the filter.
**Supplemental Figure S3**. Principal Component Analysis on the RNA‐seq data coloured by treatment and shaped by organ (**A**) or BioProject (**B**). Pearson correlation matrices between PCs and biological factors (**C**). Correlations are reported as r^2^ values. The number of asterisks indicates the statistical significance of the correlation (*: p‐value ≤ 0.05, **: p‐value ≤ 0.01, ***: p‐value ≤ 0.001).
**Supplemental Figure S4**. Bar plots showing the number of DEGs, and matrices of the Jaccard indexes for up‐ and downregulated genes in *A. thaliana* (**A**), *T. aestivum* (**B**), and *S. lycopersicum* (**C**).
**Supplemental Figure S5. A**: Genes per module for the coexpression networks of leaves and roots; **B**: Enrichment plot of the modules of the coexpression networks of leaves and roots.

**Supplemental Table S1**. List of the drought‐focused RNA‐seq studies re‐processed in this work.
**Supplemental Table S2**. List of DEGs obtained in integrated sets.
**Supplemental Table S3**. Biological processes enriched in the integrated sets.
**Supplemental Table S4**. Overlap of activated and suppressed biological processes identified through the functional enrichment analysis at the intra‐species (leaf ∩ root) and inter‐species level (all species combinations).
**Supplemental Table S5**. PlantConnectome drought‐related genes.
**Supplemental Table S6**. Orthogroups containing the genes from *Arabidopsis thaliana*, *Solanum lycopersicum* and *Triticum aestivum*.
**Supplemental Table S7**. Orthology analysis statistics.
**Supplemental Table S8**. Cross‐species conserved DEGs, with corresponding orthogroup, description and type of expression in the three species. The description was retrieved from Ensembl Plants.
**Supplemental Table S9**. Percentages of inter‐species overlaps between differentially expressed orthogroups, divided between leaves and roots.
**Supplemental Table S10**. Genes (with related additional information) belonging to the leaf black module and to the root darkorange module of the coexpression network. Information on transcription factor family membership, PlantConnectome and GO “response to water” annotation, and cross‐species conservation is included.

## Data Availability

All data, Supporting Information tables, and code used in this study are available at GitHub (https://github.com/Fra2902/Cross‐species‐transcriptomics‐on‐stressed‐plants.git) and Zenodo (https://doi.org/10.5281/zenodo.21820462).
