# Supplementary material for: Cross‐species transcriptomics uncovers shared responses to water deficiency in plants
Source: Plant Genome. 2026 Oct 8;19(4):e70311. doi: 10.1002/tpg2.70311 (PMC13650176; doi:10.1002/tpg2.70311)
Supplement: Supplementary file 1 [file TPG2-19-e70311-s001.pdf]

CROSS-SPECIES TRANSCRIPTOMICS UNCOVERS SHARED RESPONSES TO WATER DEFICIENCY IN PLANTS

Francesco Punzi, Diana Lucia Zuluaga, Qiao Wen Tan, Marek Mutwil, Gabriella Sonnante, Pasquale Luca Curci

Eight pages, five figures

Figure S1

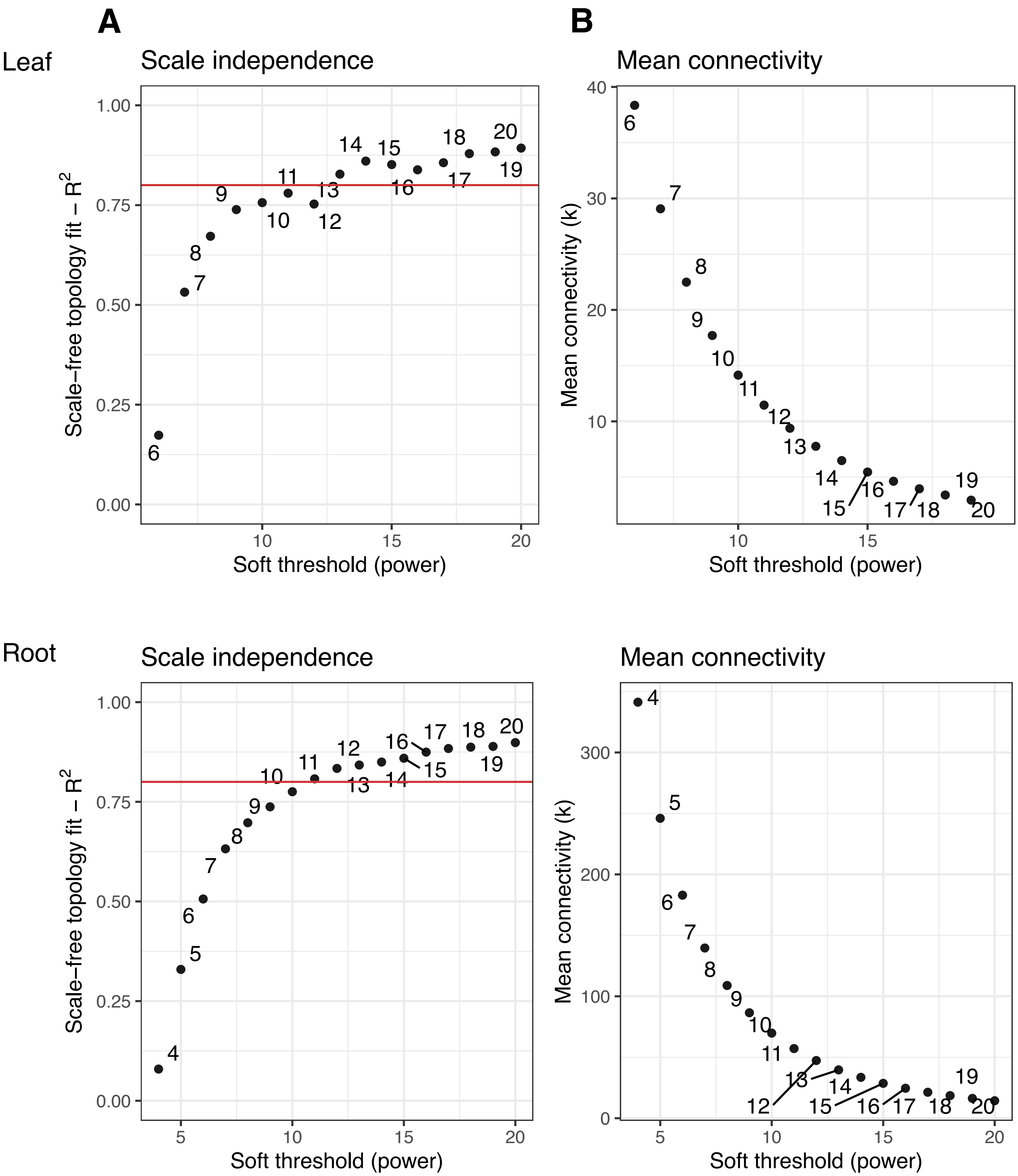

Figure S1. Plots showing the �power on the x-axis and the scale free topology R^2 (**A**) and k mean connectivity (**B**) on the y-axis, for leaves and roots.

Figure S2

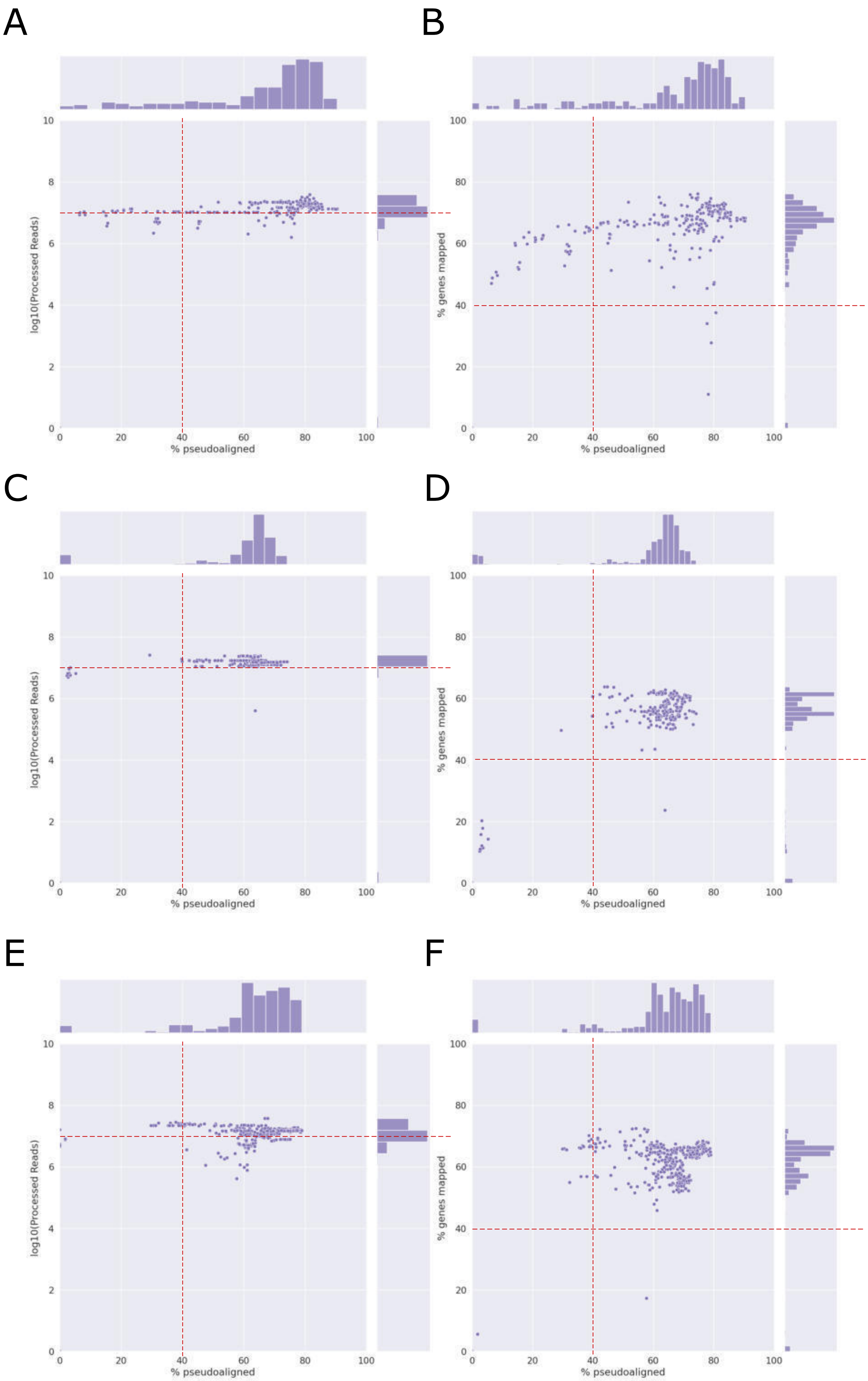

Figure S2. Alignment statistics for *A. thaliana* (**A-B**), *T. aestivum* (**C-D**) and *S. lycopersicum* (**E-F**). The red dotted lines indicate the utilised thresholds, i.e read p_pseudoaligned_threshold ≥ 40, log10(reads_pseudoaligned_threshold) ≥ 7, p_genes_mapped_threshold ≥ 40. The top-right area contains the the samples that passed the filter.

Figure S3

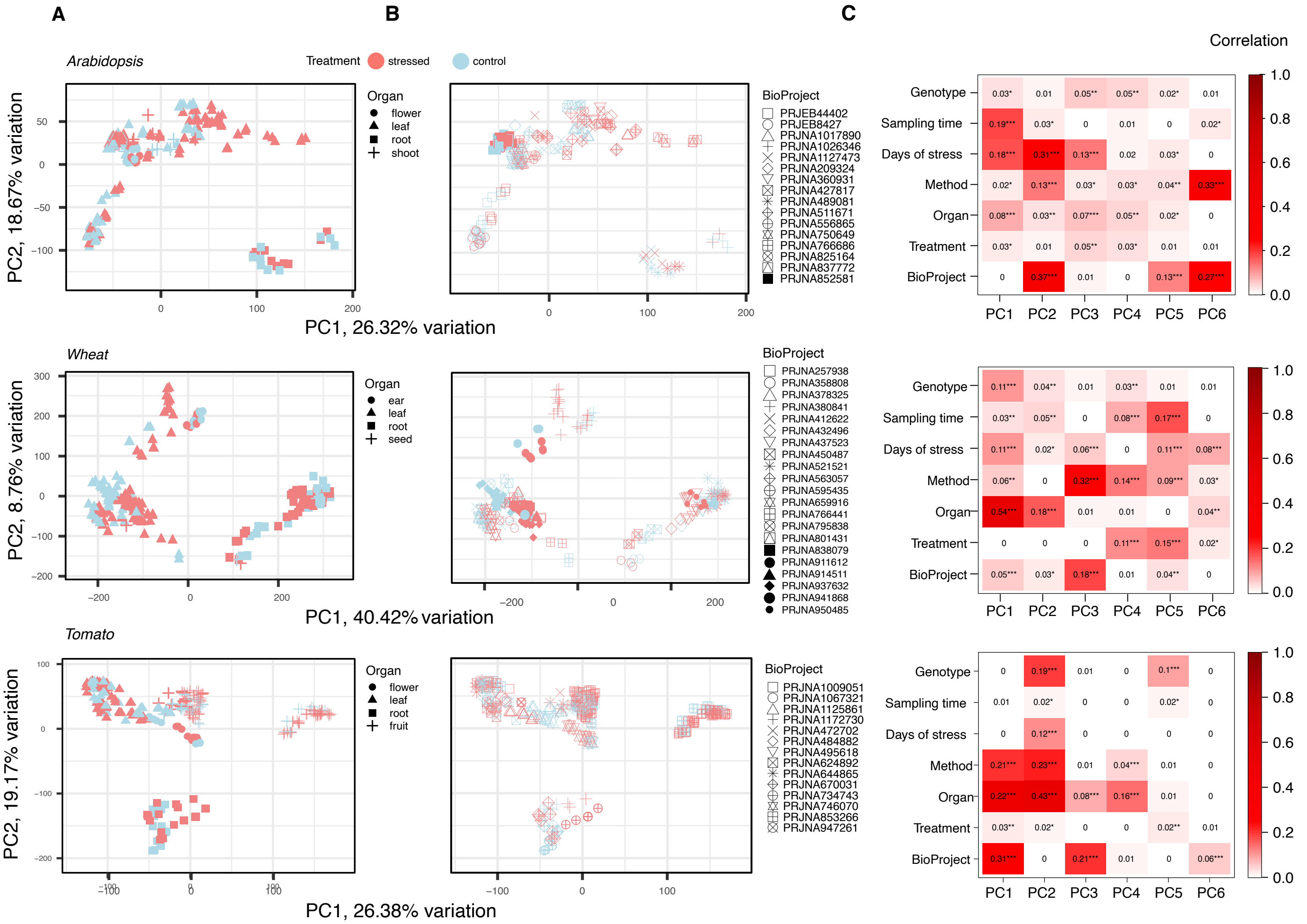

Figure S3. Principal Component Analysis on the RNA-seq data coloured by treatment and shaped by organ (**A**) or BioProject (**B**). Pearson correlation matrices between PCs and biological factors (**C**). Correlations are reported as R^2 values. The number of asterisks indicates the statistical significance of the correlation (*: p-value ≤ 0.05, **: p-value ≤ 0.01, ***: p-value ≤ 0.001).

Figure S4

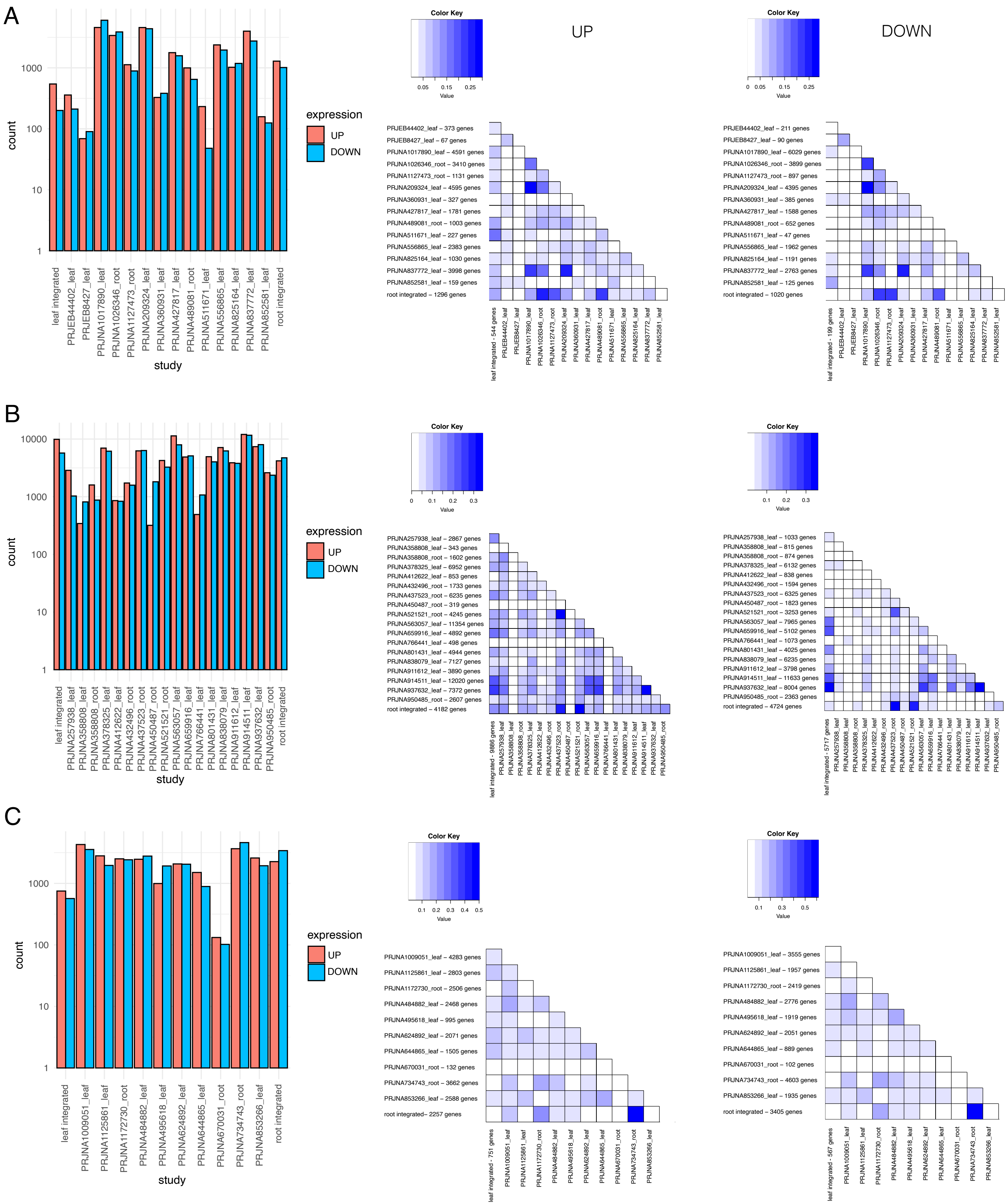

Figure S4. Bar plots showing the number of DEGs, and matrices of the Jaccard indexes for up- and downregulated genes in *A. thaliana* (**A**), *T. aestivum* (**B**) *and S. lycopersicum* (**C**) for integrated bioproject and single bioproject sets.

Figure S5

A

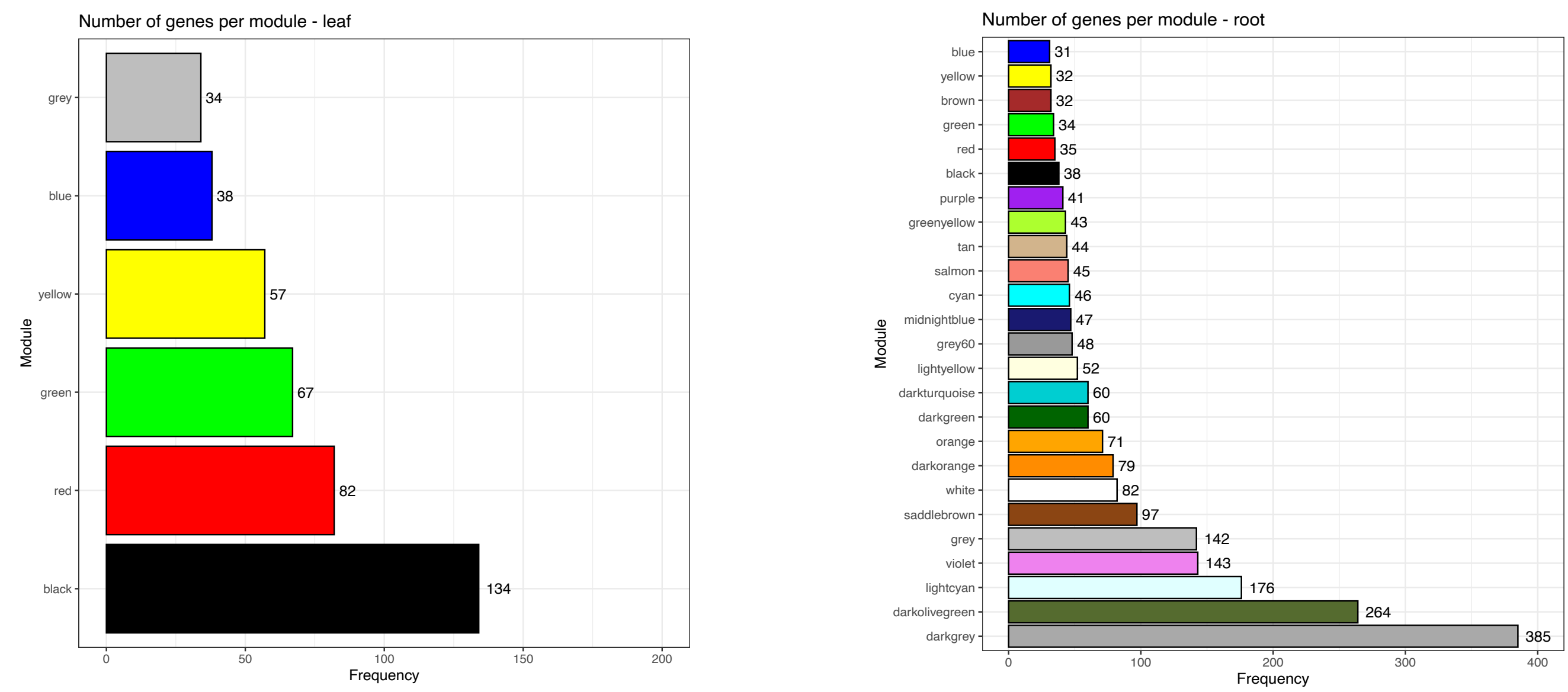

Number of genes per module - leaf
grey
blue
yellow
green
red
black
34
38
57
67
82
134
Module
0
50
100
150
200
Frequency
Number of genes per module - root
blue
yellow
brown
green
red
black
purple
greenyellow
tan
salmon
cyan
midnightblue
grey60
lightyellow
darkturquoise
darkgreen
orange
darkorange
white
saddlebrown
grey
violet
lightcyan
darkolivegreen
darkgrey
31
32
32
34
35
38
41
43
44
45
46
47
48
52
60
60
71
79
82
97
142
143
176
264
385
Module
0
100
200
300
400
Frequency

B

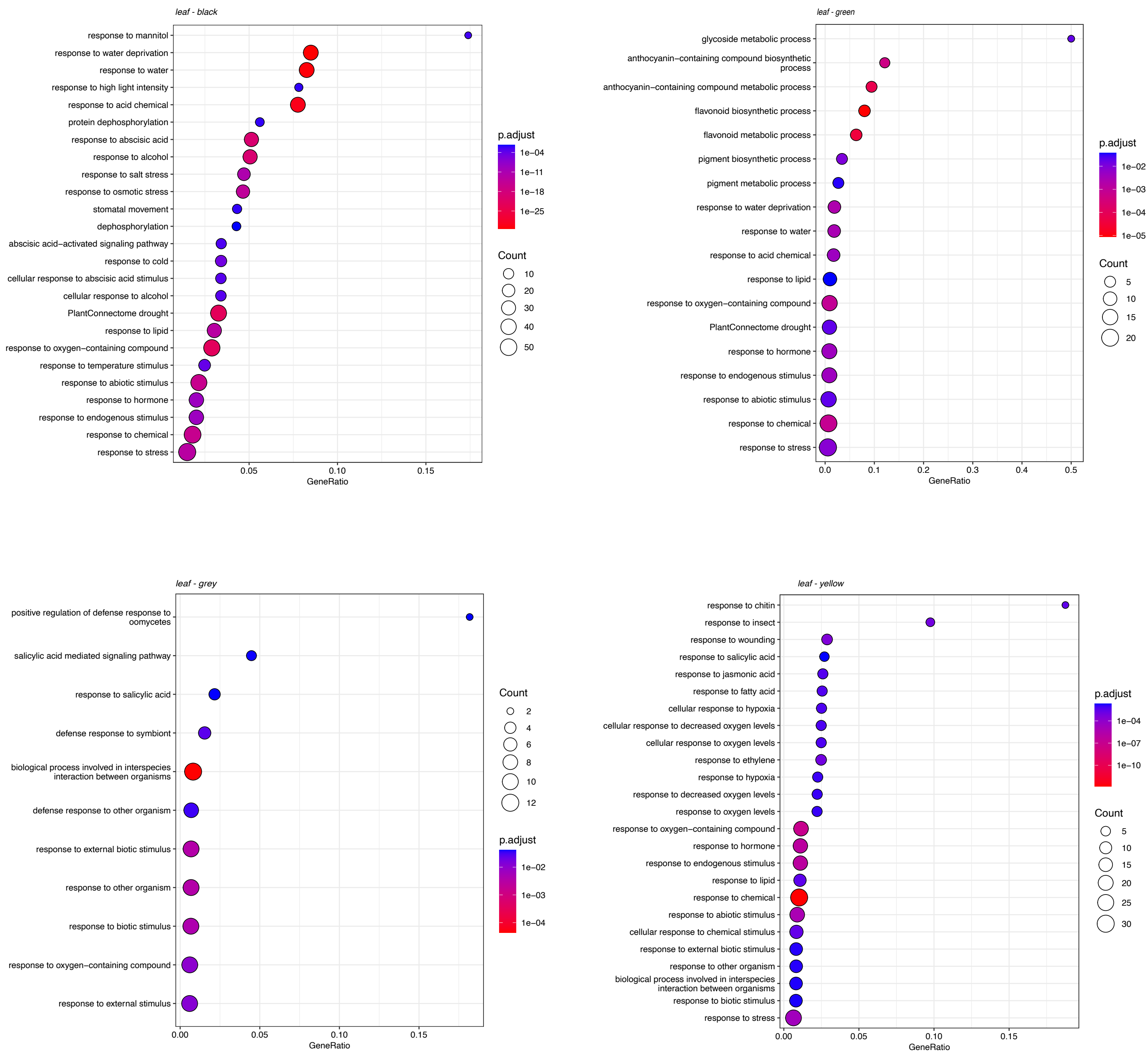

leaf - black
response to mannitol
response to water deprivation
response to water
response to high light intensity
response to acid chemical
protein dephosphorylation
response to abscisic acid
response to alcohol
response to salt stress
response to osmotic stress
stomatal movement
dephosphorylation
abscisic acid−activated signaling pathway
response to cold
cellular response to abscisic acid stimulus
cellular response to alcohol
PlantConnectome drought
response to lipid
response to oxygen−containing compound
response to temperature stimulus
response to abiotic stimulus
response to hormone
response to endogenous stimulus
response to chemical
response to stress
0.05
0.10
0.15
GeneRatio
p.adjust
1e−04
1e−11
1e−18
1e−25
Count
10
20
30
40
50
leaf - green
glycoside metabolic process
anthocyanin−containing compound biosynthetic process
anthocyanin−containing compound metabolic process
flavonoid biosynthetic process
flavonoid metabolic process
pigment biosynthetic process
pigment metabolic process
response to water deprivation
response to water
response to acid chemical
response to lipid
response to oxygen−containing compound
PlantConnectome drought
response to hormone
response to endogenous stimulus
response to abiotic stimulus
response to chemical
response to stress
0.0
0.1
0.2
0.3
0.4
0.5
GeneRatio
p.adjust
1e−02
1e−03
1e−04
1e−05
Count
5
10
15
20
leaf - grey
positive regulation of defense response to oomycetes
salicylic acid mediated signaling pathway
response to salicylic acid
defense response to symbiont
biological process involved in interspecies interaction between organisms
defense response to other organism
response to external biotic stimulus
response to other organism
response to biotic stimulus
response to oxygen−containing compound
response to external stimulus
0.00
0.05
0.10
0.15
GeneRatio
Count
2
4
6
8
10
12
p.adjust
1e−02
1e−03
1e−04
leaf - yellow
response to chitin
response to insect
response to wounding
response to salicylic acid
response to jasmonic acid
response to fatty acid
cellular response to hypoxia
cellular response to decreased oxygen levels
cellular response to oxygen levels
response to ethylene
response to hypoxia
response to decreased oxygen levels
response to oxygen levels
response to oxygen−containing compound
response to hormone
response to endogenous stimulus
response to lipid
response to chemical
response to abiotic stimulus
cellular response to chemical stimulus
response to external biotic stimulus
response to other organism
biological process involved in interspecies interaction between organisms
response to biotic stimulus
response to stress
0.00
0.05
0.10
0.15
GeneRatio
p.adjust
1e−04
1e−07
1e−10
Count
5
10
15
20
25
30

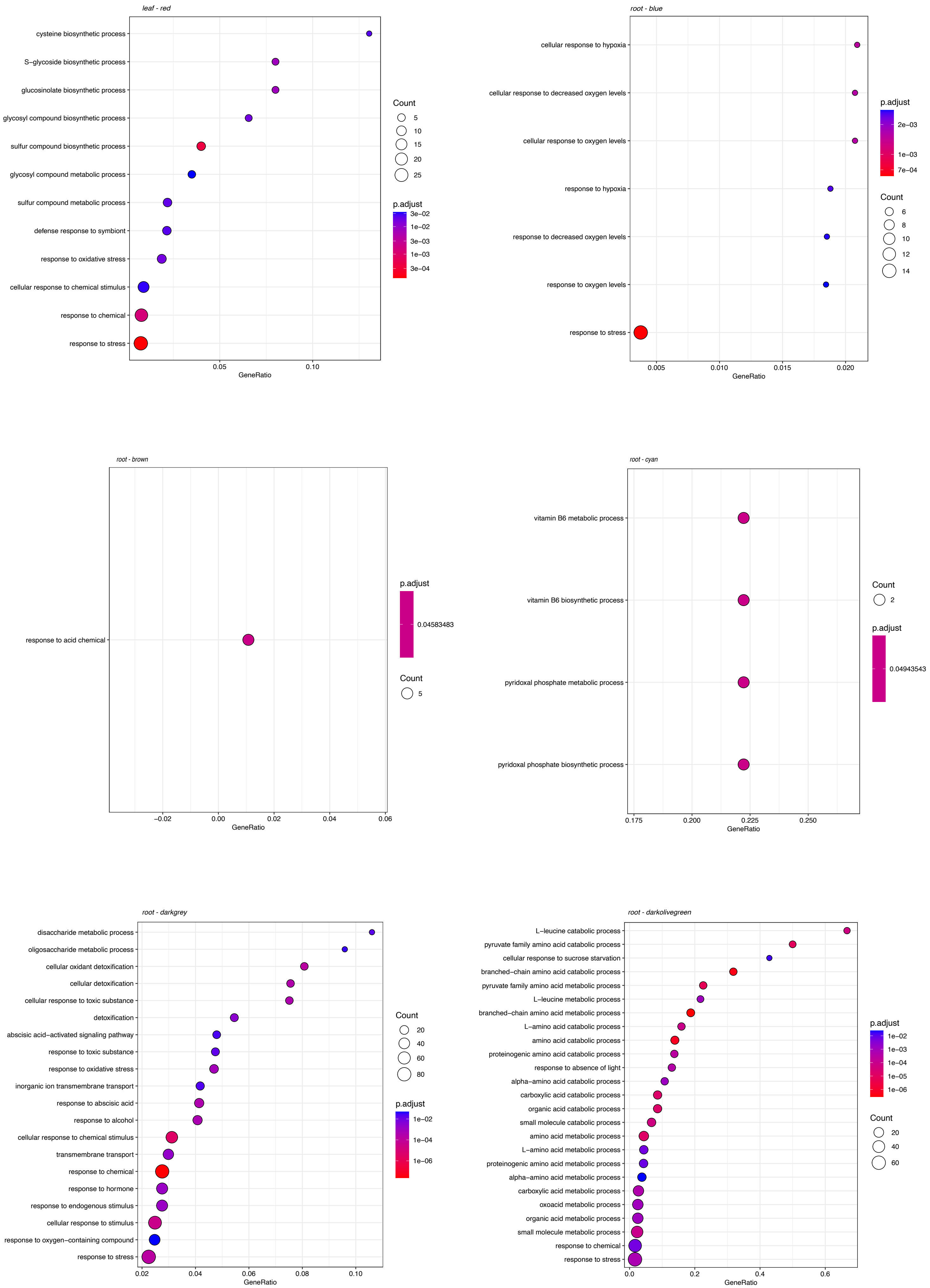
leaf - red
cysteine biosynthetic process
S−glycoside biosynthetic process
glucosinolate biosynthetic process
glycosyl compound biosynthetic process
sulfur compound biosynthetic process
glycosyl compound metabolic process
sulfur compound metabolic process
defense response to symbiont
response to oxidative stress
cellular response to chemical stimulus
response to chemical
response to stress
0.05
0.10
GeneRatio
Count
5
10
15
20
25
p.adjust
3e−02
1e−02
3e−03
1e−03
3e−04
root - blue
cellular response to hypoxia
cellular response to decreased oxygen levels
cellular response to oxygen levels
response to hypoxia
response to decreased oxygen levels
response to oxygen levels
response to stress
0.005
0.010
0.015
0.020
GeneRatio
p.adjust
2e−03
1e−03
7e−04
Count
6
8
10
12
14
root - brown
response to acid chemical
−0.02
0.00
0.02
0.04
0.06
GeneRatio
p.adjust
0.04583483
Count
5
root - cyan
vitamin B6 metabolic process
vitamin B6 biosynthetic process
pyridoxal phosphate metabolic process
pyridoxal phosphate biosynthetic process
0.175
0.200
0.225
0.250
GeneRatio
Count
2
p.adjust
0.04943543
root - darkgrey
disaccharide metabolic process
oligosaccharide metabolic process
cellular oxidant detoxification
cellular detoxification
cellular response to toxic substance
detoxification
abscisic acid−activated signaling pathway
response to toxic substance
response to oxidative stress
inorganic ion transmembrane transport
response to abscisic acid
response to alcohol
cellular response to chemical stimulus
transmembrane transport
response to chemical
response to hormone
response to endogenous stimulus
cellular response to stimulus
response to oxygen−containing compound
response to stress
0.02
0.04
0.06
0.08
0.10
GeneRatio
Count
20
40
60
80
p.adjust
1e−02
1e−04
1e−06
root - darkolivegreen
L−leucine catabolic process
pyruvate family amino acid catabolic process
cellular response to sucrose starvation
branched−chain amino acid catabolic process
pyruvate family amino acid metabolic process
L−leucine metabolic process
branched−chain amino acid metabolic process
L−amino acid catabolic process
amino acid catabolic process
proteinogenic amino acid catabolic process
response to absence of light
alpha−amino acid catabolic process
carboxylic acid catabolic process
organic acid catabolic process
small molecule catabolic process
amino acid metabolic process
L−amino acid metabolic process
proteinogenic amino acid metabolic process
alpha−amino acid metabolic process
carboxylic acid metabolic process
oxoacid metabolic process
organic acid metabolic process
small molecule metabolic process
response to chemical
response to stress
0.0
0.2
0.4
0.6
GeneRatio
p.adjust
1e−02
1e−03
1e−04
1e−05
1e−06
Count
20
40
60

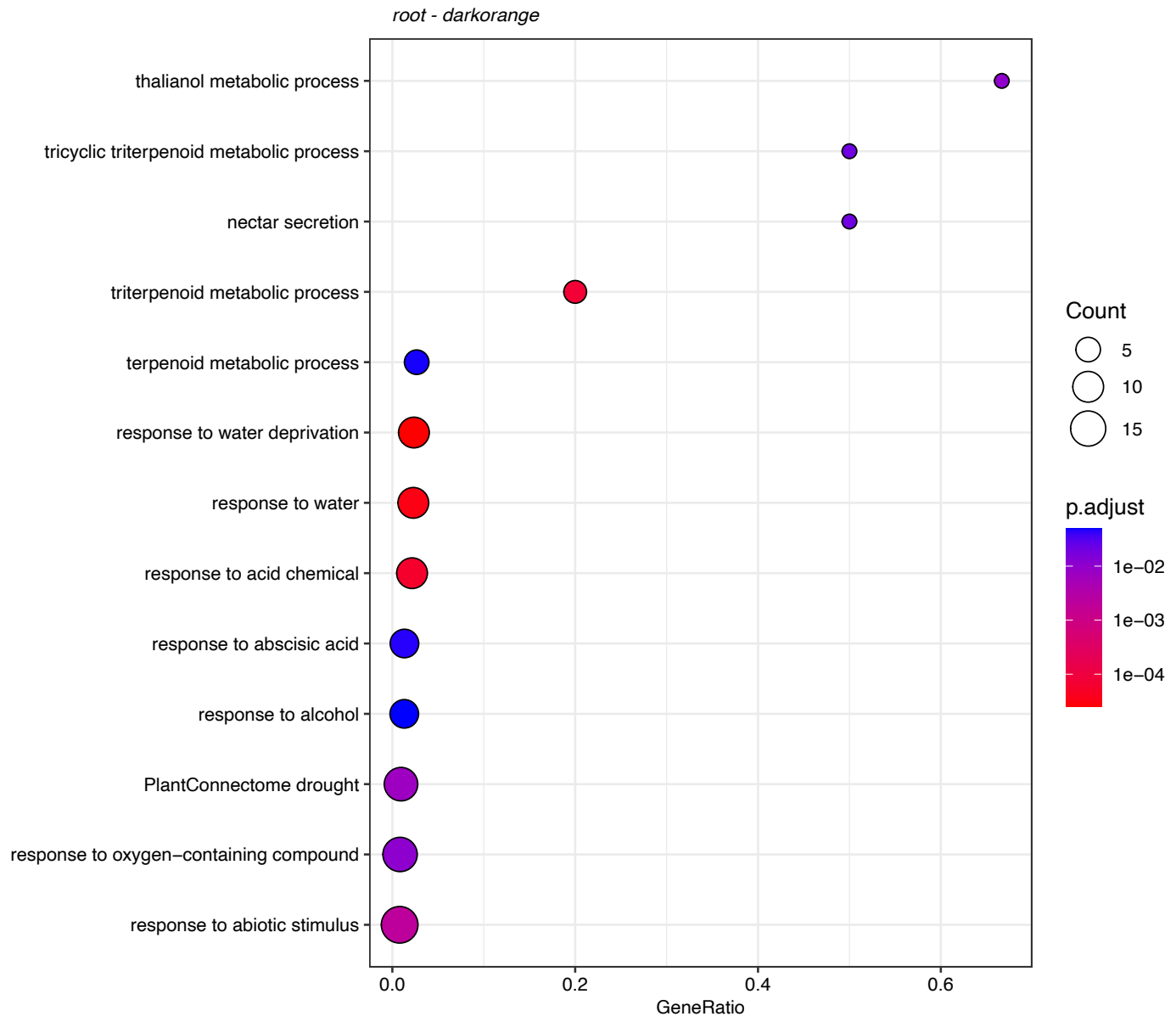
root - darkorange
thalianol metabolic process
tricyclic triterpenoid metabolic process
nectar secretion
triterpenoid metabolic process
terpenoid metabolic process
response to water deprivation
response to water
response to acid chemical
response to abscisic acid
response to alcohol
PlantConnectome drought
response to oxygen−containing compound
response to abiotic stimulus
0.0
0.2
0.4
0.6
GeneRatio
Count
5
10
15
p.adjust
1e−02
1e−03
1e−04

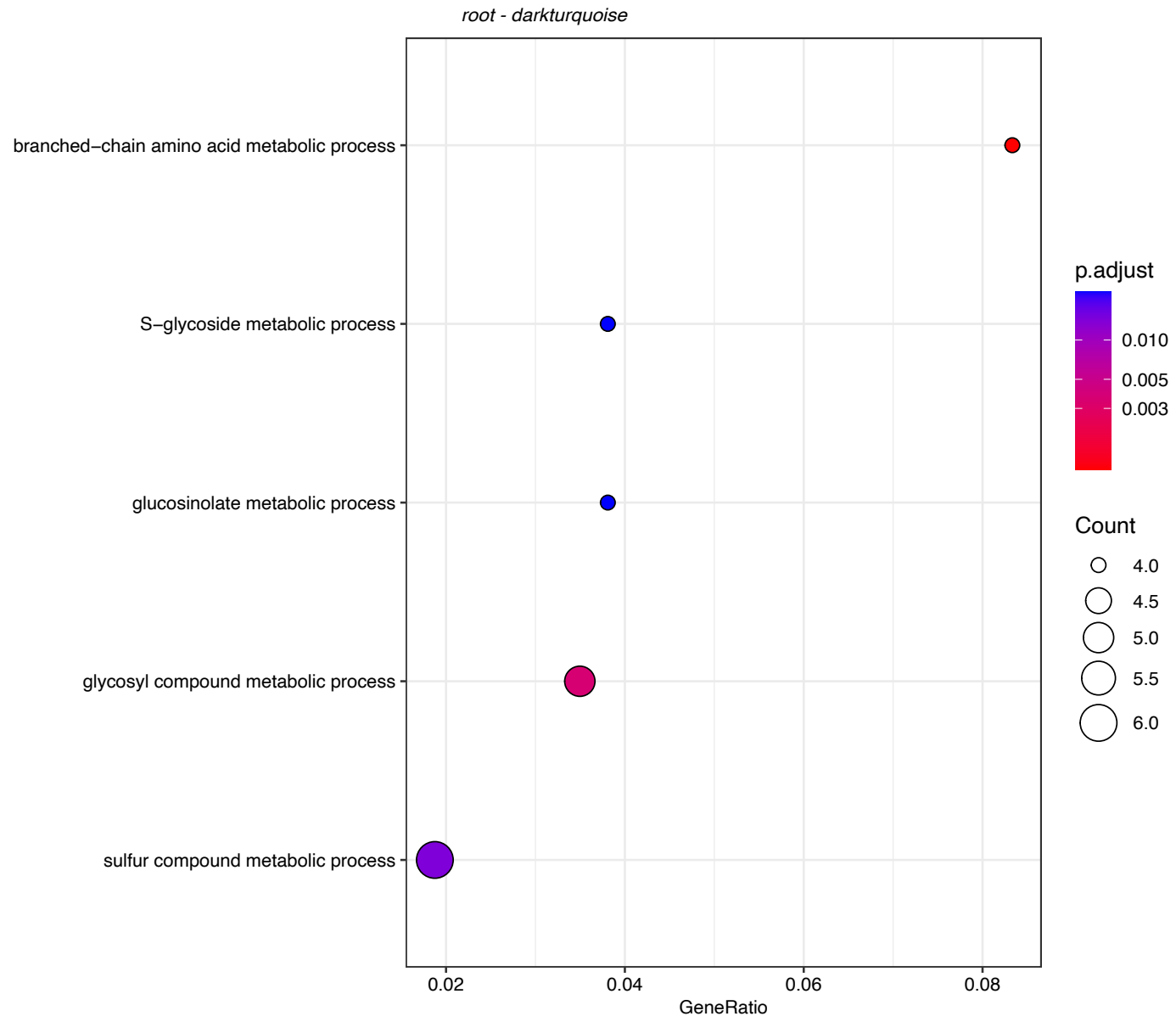
root - darkturquoise
branched−chain amino acid metabolic process
S−glycoside metabolic process
glucosinolate metabolic process
glycosyl compound metabolic process
sulfur compound metabolic process
0.02
0.04
0.06
0.08
GeneRatio
p.adjust
0.010
0.005
0.003
Count
4.0
4.5
5.0
5.5
6.0

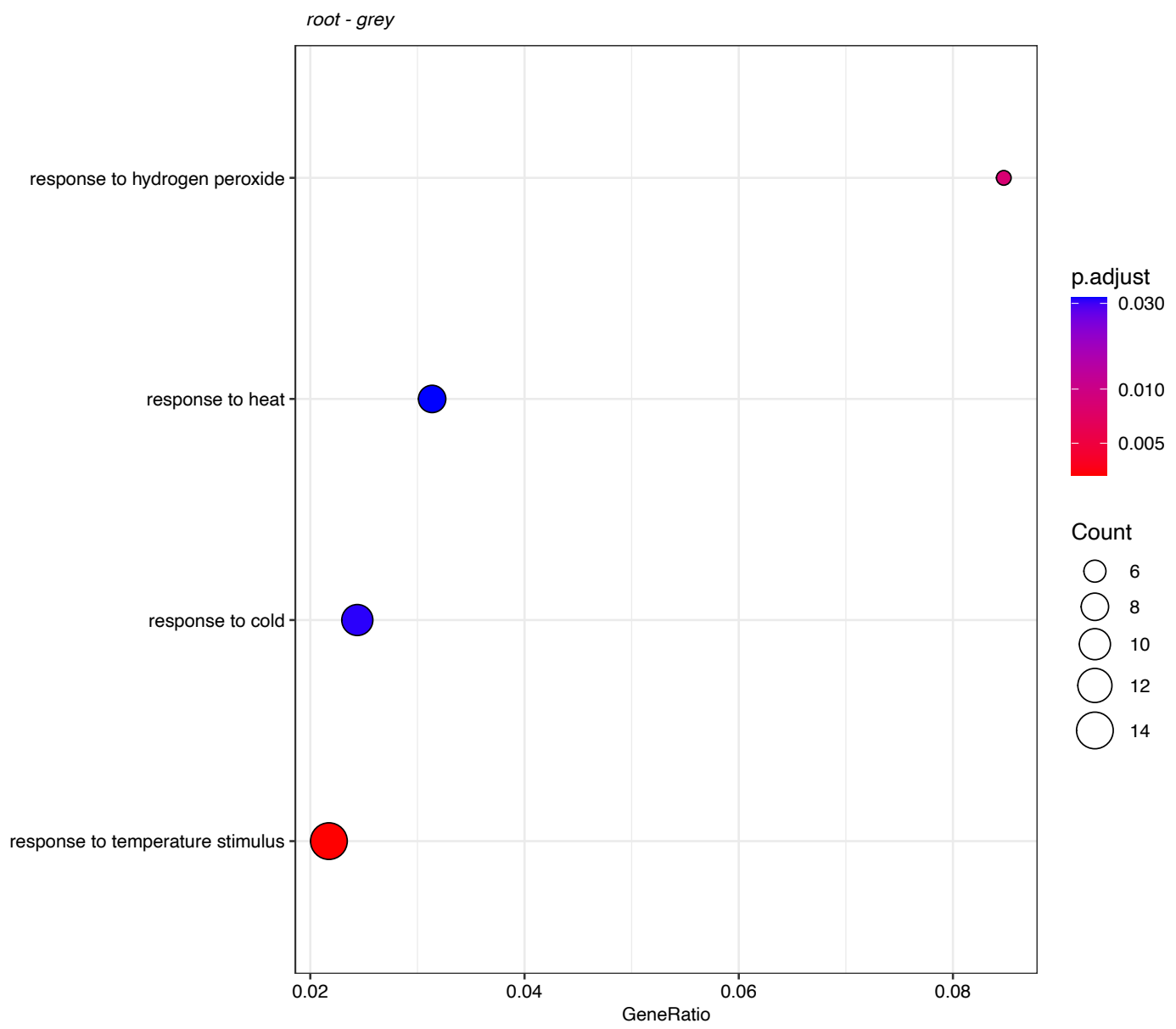
root - grey
response to hydrogen peroxide
response to heat
response to cold
response to temperature stimulus
0.02
0.04
0.06
0.08
GeneRatio
p.adjust
0.030
0.010
0.005
Count
6
8
10
12
14

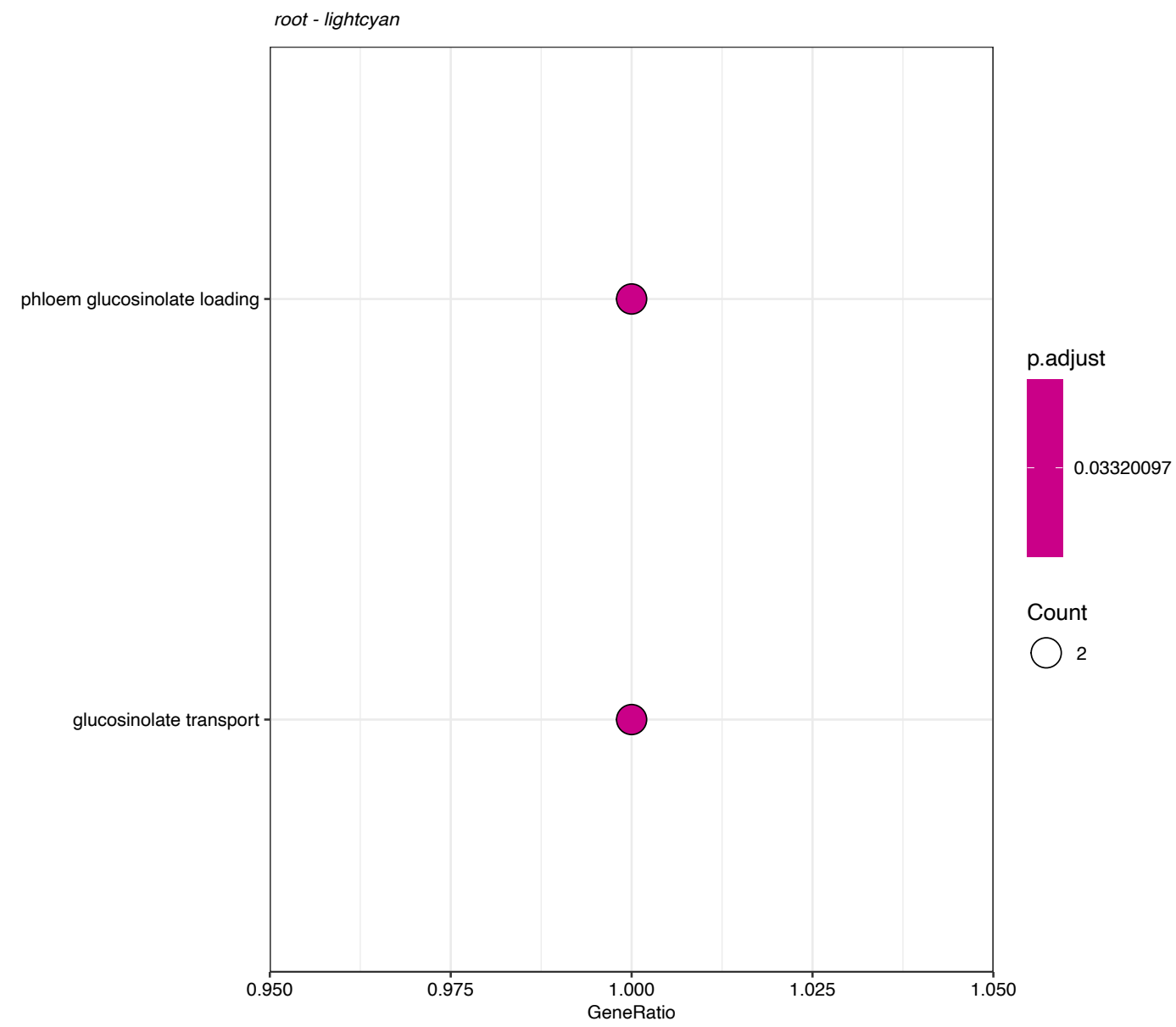
root - lightcyan
phloem glucosinolate loading
glucosinolate transport
0.950
0.975
1.000
1.025
1.050
GeneRatio
p.adjust
0.03320097
Count
2

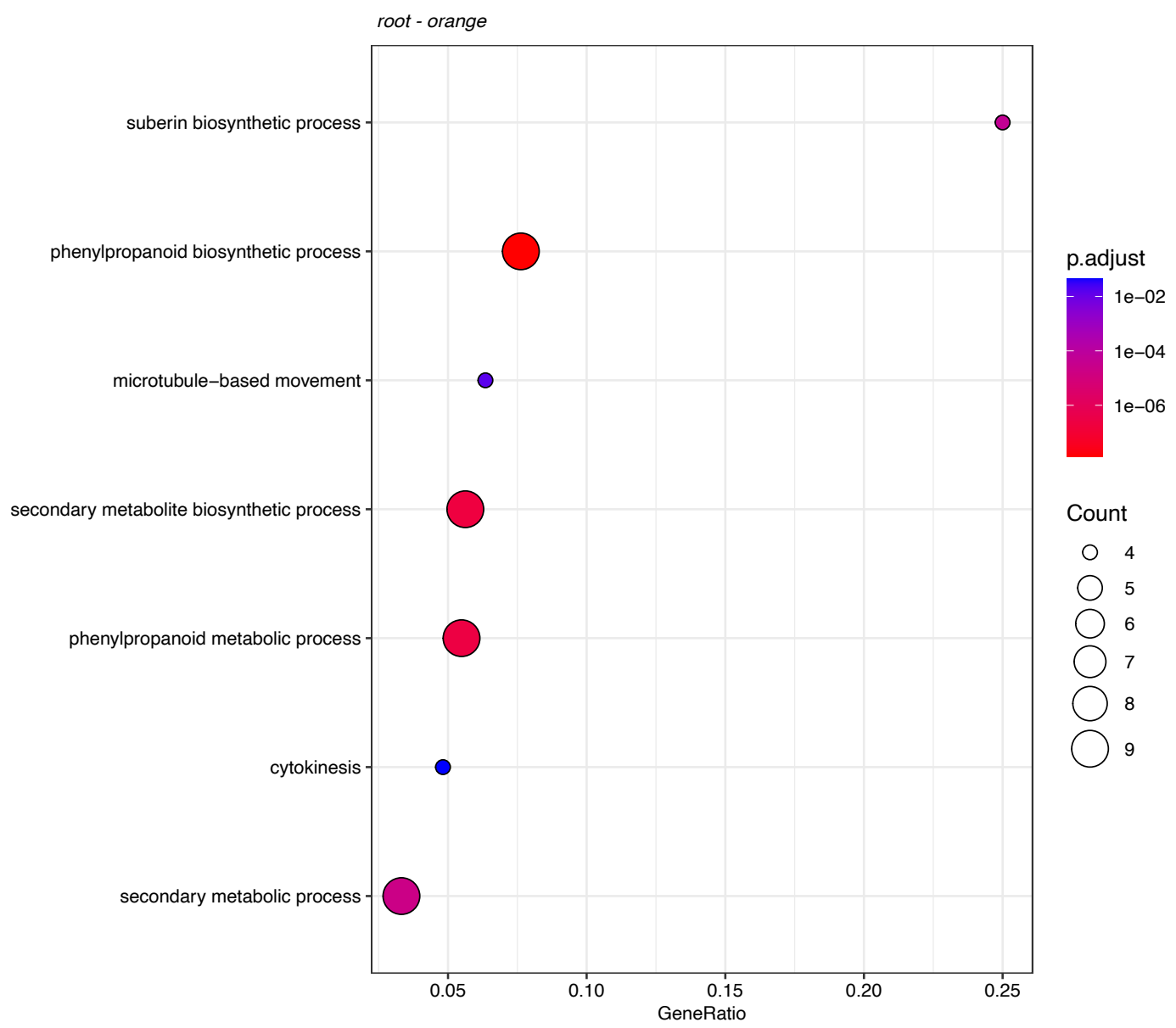
root - orange
suberin biosynthetic process
phenylpropanoid biosynthetic process
microtubule−based movement
secondary metabolite biosynthetic process
phenylpropanoid metabolic process
cytokinesis
secondary metabolic process
0.05
0.10
0.15
0.20
0.25
GeneRatio
p.adjust
1e−02
1e−04
1e−06
Count
4
5
6
7
8
9

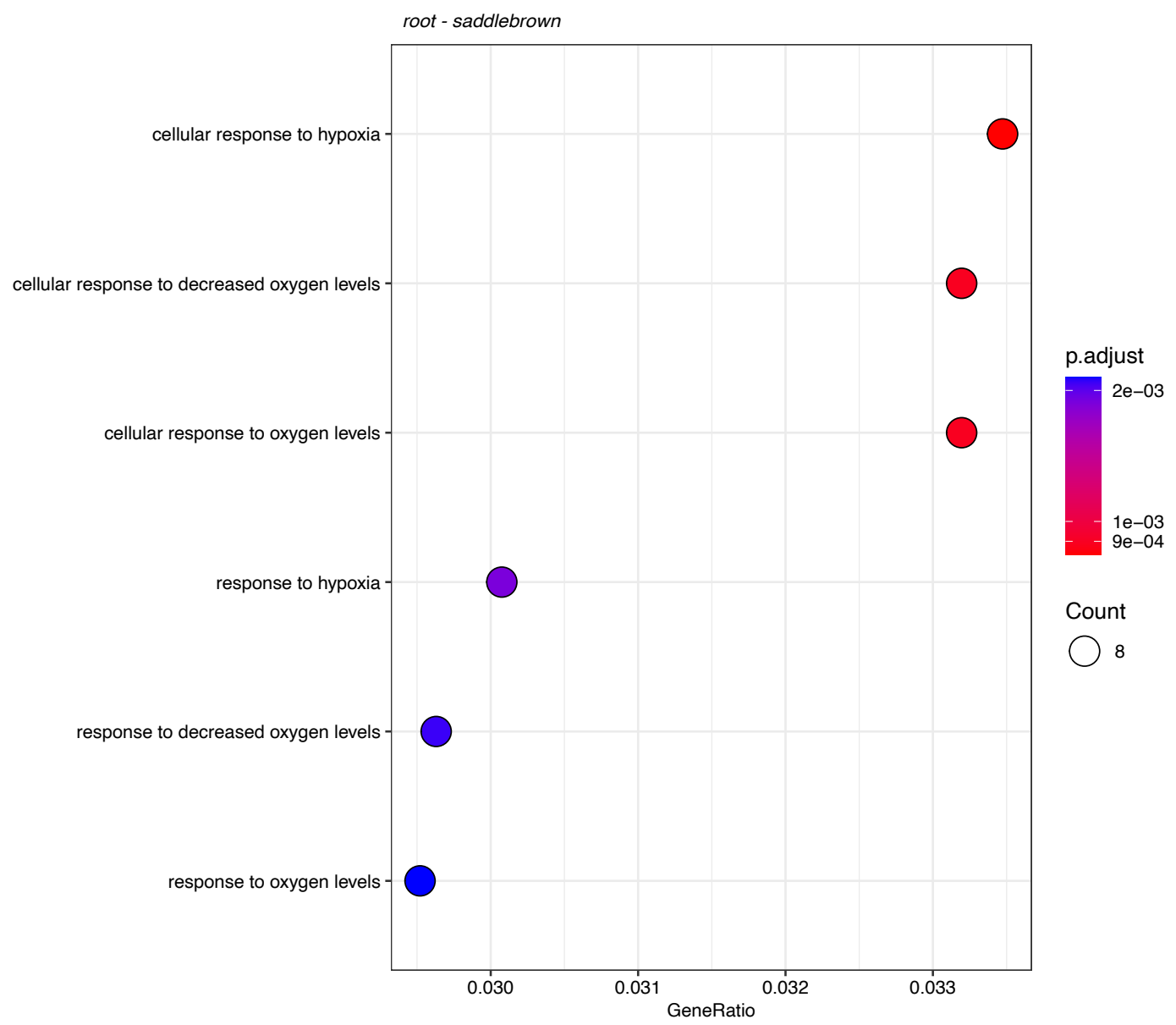
root - saddlebrown
cellular response to hypoxia
cellular response to decreased oxygen levels
cellular response to oxygen levels
response to hypoxia
response to decreased oxygen levels
response to oxygen levels
0.030
0.031
0.032
0.033
GeneRatio
p.adjust
2e−03
1e−03
9e−04
Count
8

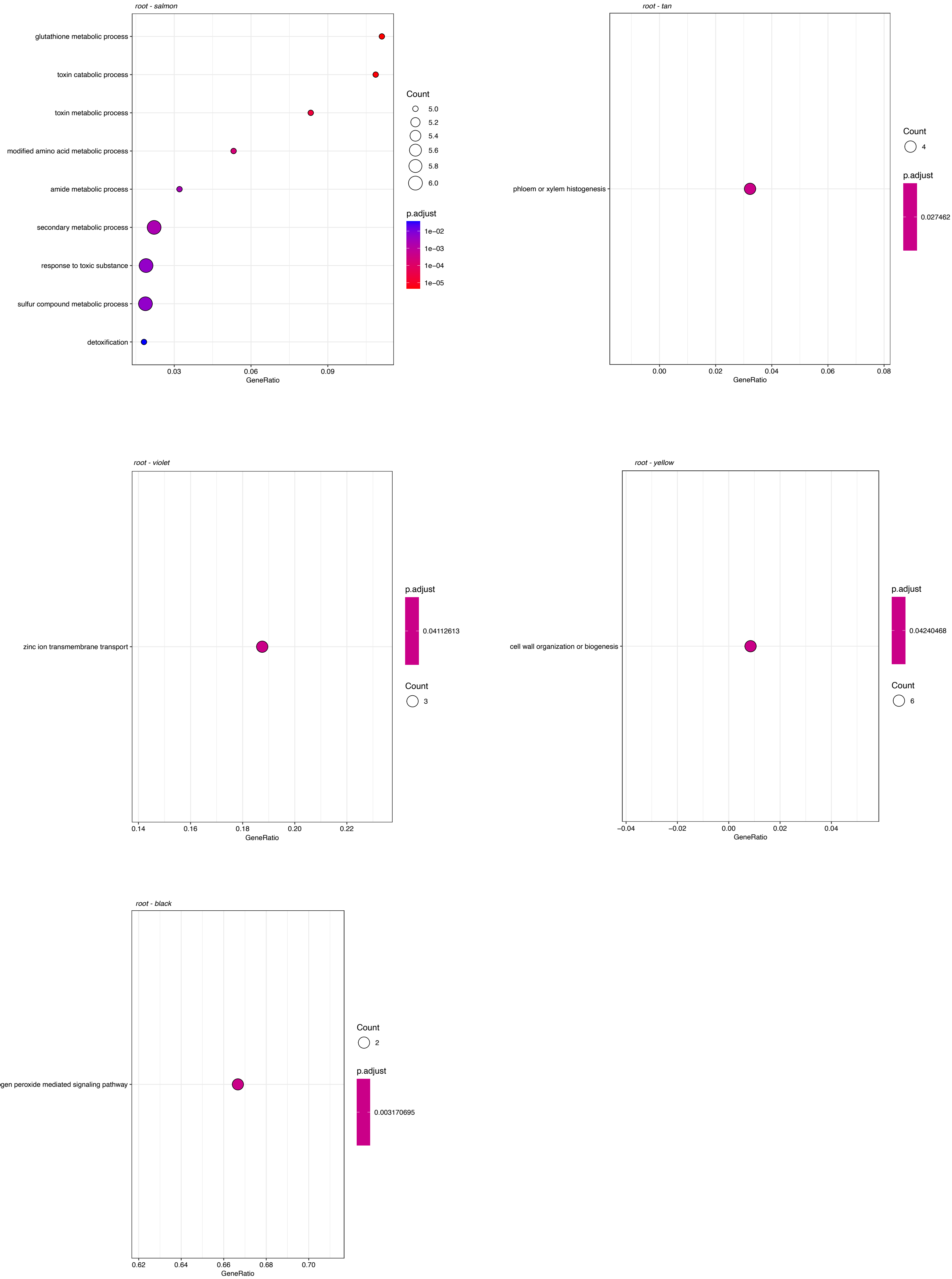

Figure S5. A: Genes per module for the coexpression networks of leaves and roots; **B:** Enrichment plot of the modules of the coexpression networks of leaves and roots.
