# Supplementary material for: Cross‐species transcriptomics uncovers shared responses to water deficiency in plants
Source: Plant Genome. 2026 Oct 8;19(4):e70311. doi: 10.1002/tpg2.70311 (PMC13650176; doi:10.1002/tpg2.70311)
Supplement: Supplementary file 2 [file TPG2-19-e70311-s002.pdf]

Supplemental material and code availability

Since the files are momentarily private, code and supplemental tables are

accessible via: https://zenodo.org/records/21820462?token=eyJhbGciOiJIUzUxMiJ9.eyJpZCI6ImFjNDcyNWQyLWMyMGItNDAxNC1hZjI5LWI0NzZmN2FlZGYyNiIsImRhdGEiOnt9LCJyYW5kb20iOiJkMzY0NThjMTZiODE3MDA1ZWM4OGIwZDY0MTE1NzA0ZSJ9.aD22_YHmobWSbLhbM9TBoXToMfFtR19O8lFnTr8xoQbC7dxQm_bZ8f718X36FVyItxfQUE5SalWzTeneIciKrQ

Please verify, within the “Versions” panel (right side of the webpage) that you’re downloading **Version v3** 10.5281/zenodo.21820462

File: Data.zip

md5:e66e3c99a7565d0c586fc2e7e4735a3a

Size: 225.3 MB
